# Supplementary material for: Improvements in blood and fitness tracker biomarkers in a longitudinal real-world cohort of digital health platform users
Source: PLOS Digit Health. 2026 Mar 24;5(3):e0001271. doi: 10.1371/journal.pdig.0001271 (PMC13012459; doi:10.1371/journal.pdig.0001271)
Supplement: S1 Table — (PDF) [file pdig.0001271.s001.pdf]

Table S1a. Blood biomarker sample size and summary statistics

Biomarker abbreviation	Biomarker name	Unit	Paired longitudinal samples	Median	IQR	25th %	75th %	Shapiro-Wilk p
Alb	albumin	g/dL	15778	4.5	0.4	4.3	4.7	5.40E-12
ALT	alanine aminotransferase	U/L	16200	20	11	16	27	6.00E-37
APOB	apolipoprotein B	mg/dL	726	93	31	79	110	1.47E-04
AST	aspartate aminotransferase	U/L	15206	22	8	18	26	4.42E-36
B12	vitamin B12	pg/mL	12443	528.45	318	404	722	1.93E-43
Ca	calcium	mg/dL	16493	9.5	0.5	9.3	9.8	7.29E-11
CK	creatine kinase	U/L	11644	114	104	77	181	1.01E-49
Cor	cortisol	µg/dL	10650	12.9	6.8	9.8	16.6	4.57E-18
D	vitamin D	ng/mL	14091	36	20	28	48	2.76E-29
DHEAS	DHEAS	µg/dL	4088	113	96	72	168	7.17E-28
FE	iron	ug/dL	11837	109	51	85	136	6.49E-12
Fer	ferritin	ng/mL	13251	82	110	41	151	3.36E-38
Fol	folate	ng/mL	11847	15.69	7.8	12	19.8	1.18E-20
FT	free testosterone	ng/dL	7541	7.89	3.15	6.39	9.54	1.45E-23
GGT	gamma-glutamyl transpeptidase	U/L	12737	15	9	12	21	1.36E-46
Glu	fasting glucose	mg/dL	18155	89	12	83	95	3.44E-12
Hb	hemoglobin	g/dL	18054	14.5	1.7	13.7	15.4	2.46E-06
HCT	hematocrit	%	16659	43.4	4.8	41	45.8	3.98E-05
HDL-c	high-density lipoprotein cholesterol	mg/dL	17919	60	22	50	72	5.79E-19
HgbA1c	hemoglobin A1c	%	12555	5.2	0.4	5	5.4	1.51E-17
hsCRP	high sensitivity C-reactive protein	mg/L	11635	0.6	1.1	0.3	1.4	6.63E-56
K	potassium	mmol/L	14468	4.3	0.5	4.1	4.6	1.06E-13
LDL-c	low-density lipoprotein cholesterol	mg/dL	17848	111	46	89	135	4.69E-15
MCH	mean corpuscular hemoglobin	pg	16347	30.5	1.8	29.6	31.4	3.42E-20

Biomarker abbreviation	Full Name	Unit	Paired longitudinal samples	Median	IQR	25th %	75th %	Shapiro-Wilk p
MCHC	mean corpuscular hemoglobin concentration	g/dL	16206	33.5	1.2	32.9	34.1	5.66E-14
MCV	mean corpuscular volume	fL	16511	91	5.6	88.2	93.8	1.53E-03
Mg	magnesium	mg/dL	11951	2.1	0.2	2	2.2	1.70E-20
MPV	mean platelet volume	fL	13178	10.5	1.37	9.8	11.17	5.69E-06
Na	sodium	mmol/L	15789	139	3	138	141	1.28E-17
PLT	platelets	thousands/uL	16493	234	65	203	268	2.39E-12
RBC	red blood cells	10^6/µL	16605	4.78	0.62	4.47	5.09	6.90E-07
RBC_Mg	red blood cell magnesium	mg/dL	8285	5	0.9	4.6	5.5	1.16E-07
RDW	red cell distribution width	%	15748	12.7	1	12.2	13.2	2.80E-26
SHBG	sex-hormone binding globulin	nmol/L	11348	50	36	36	72	1.52E-36
TC	total cholesterol	mg/dL	18095	191	50	167	217	1.32E-12
Tes	testosterone	ng/dL	12841	444	583	35	618	7.74E-31
Tg	triglycerides	mg/dL	17978	76	46	58	104	2.82E-39
TIBC	total iron-binding capacity	ug/dL	10718	324	58.91	296.09	355	2.70E-14
TS	transferrin saturation	%	10889	34	16	26	42	1.04E-12
