# Supplementary material for: Improvements in blood and fitness tracker biomarkers in a longitudinal real-world cohort of digital health platform users
Source: PLOS Digit Health. 2026 Mar 24;5(3):e0001271. doi: 10.1371/journal.pdig.0001271 (PMC13012459; doi:10.1371/journal.pdig.0001271)
Supplement: S2 Table — (PDF) [file pdig.0001271.s002.pdf]

Table S1b. Physiological biomarker & PGS sample size and summary statistics

Fitness Tracker Biomarker	Unit	Users with linked blood draw	Median	IQR	25th %	75th %	Shapiro-Wilk p
daily active calories burned	kcals	2455	887	511.96	671.62	1183.58	3.63E-27
nightly deep sleep	seconds	2663	3962.86	1693.24	3151.76	4845	1.85E-26
Calculated polygenic scores (PGS)	NA	7488	NA	NA	NA	NA	NA
nightly REM sleep	seconds	2663	5332.86	2220.57	4264.43	6485	5.77E-14
resting heart rate	beats/min	2436	54.37	9.7	49.86	59.55	6.89E-17
nightly sleep duration	seconds	2541	24162.35	5162.86	21600	26762.86	2.69E-10
daily step count	steps	1640	11065.85	4682.12	8993.3	13675.42	1.04E-15
maximal oxygen consumption (vo2 max)	mL/kg/min	1610	46.89	8.71	42.29	51	1.19E-09

* For wearable data to be linked to a blood draw, it must be collected within the 30 days prior to that draw.
