# Supplementary material for: Improvements in blood and fitness tracker biomarkers in a longitudinal real-world cohort of digital health platform users
Source: PLOS Digit Health. 2026 Mar 24;5(3):e0001271. doi: 10.1371/journal.pdig.0001271 (PMC13012459; doi:10.1371/journal.pdig.0001271)
Supplement: S3 Table — (PDF) [file pdig.0001271.s003.pdf]

Table S2a. Users with higher than optimal baseline biomarker levels: baseline vs. follow-up

Biomarker	Mann-Whitney p	Draw 1 median(IQR)	Draw 2 median(IQR)	Unit	% users improved*	% users optimized*	Paired samples
NEUT	8.10E-19	8875.5(1500)	4033(3100)	cells/µL	77.30%	75.80%	66
BASOS	0.001	300(130)	60(72)	cells/µL	72.70%	72.70%	11
D	2.70E-17	102(1)	66(26)	ng/mL	73.40%	71.50%	158
MONOS	2.80E-15	1040(140)	600(340)	cells/µL	71.40%	71.40%	77
Alb	4.10E-69	5.2(0.1)	4.9(0.3)	g/dL	71.70%	71.10%	318
K	3.30E-211	5(0.2)	4.5(0.5)	mmol/L	79.50%	64.30%	1162
EOS	2.40E-43	621(170)	370(290)	cells/µL	65.70%	63.90%	277
Hb	1.30E-18	17.2(1.4)	16.2(1.5)	g/dL	64.70%	62.40%	354
HCT	2.10E-45	50.2(4.8)	46.8(4.7)	%	59.90%	59.60%	862
MCV	9.00E-49	101(1.5)	97.6(5.3)	fL	59.10%	54.40%	467
TIBC	8.60E-34	451(24)	402(58)	ug/dL	53.90%	53.90%	293
LYMPHS	7.40E-05	4390(950)	3640.5(2100)	cells/µL	54.70%	52.80%	53
LDL-c	6.30E-109	149(27)	141(37)	mg/dL	20.40%	5.30%	5128
WBC	2.60E-205	7.5(1.4)	6.5(2)	thousands/uL	86.00%	46.50%	2778
Tes	1.70E-10	81(1000)	72(750)	ng/dL	49.90%	45.90%	653
RBC_Mg	1.80E-20	6.6(0.3)	6.2(0.83)	mg/dL	45.90%	45.90%	209
RDW	7.70E-39	15.5(0.5)	14.25(2)	%	45.60%	45.30%	395
RBC	2.10E-08	5.57(0.63)	5.415(0.66)	x10E6/µL	44.50%	44.50%	411
Cor	9.10E-165	18.9(4.2)	15.6(6.9)	µg/dL	73.70%	41.00%	3027
MCH	7.30E-39	33.4(0.5)	33(1.1)	pg	40.80%	40.80%	622
CK	2.90E-120	261(160)	194(160)	U/L	76.90%	37.30%	2993
AST	6.80E-192	29(8)	26(9)	U/L	76.00%	35.40%	5054
DHEAS	3.10E-13	258(86)	207(110)	µg/dL	39.80%	35.40%	322
B12	1.10E-140	963.405(430)	787(390)	pg/mL	65.90%	32.00%	2833
MPV	5.10E-49	12(0.6)	11.8(1)	fL	74.20%	31.50%	2066
Glu	2.90E-231	95(8)	91(11)	mg/dL	74.20%	31.10%	9180
Tg	2.70E-146	113(48)	99(57)	mg/dL	76.20%	29.60%	7215
ALT	6.30E-117	28(12)	25(13)	U/L	77.40%	26.70%	7620
FE	2.10E-234	138(35)	119(51)	ug/dL	77.90%	26.70%	5660
hsCRP	1.30E-83	1.5(1.7)	1.2(1.7)	mg/L	70.70%	25.40%	5005
FT	0.051	8.836(2.6)	8.71(3.7)	ng/dL	38.30%	24.40%	209
Fer	3.00E-28	223(120)	203(120)	ng/mL	72.50%	21.40%	2469
HgbA1c	2.80E-42	5.4(0.3)	5.4(0.3)	%	79.30%	21.40%	5533
GGT	2.50E-48	22(10)	21(11)	U/L	84.00%	20.70%	5219

Biomarker	Mann-Whitney p	Draw 1 median(IQR)	Draw 2 median(IQR)	Unit	% users improved*	% users optimized*	Paired samples
TC	4.70E-60	216(34)	210(45)	mg/dL	42.20%	20.60%	9178
APOB	2.40E-10	109(20)	102(24)	mg/dL	66.30%	20.50%	404
SHBG	1.10E-18	67.16(35)	64.5(35)	nmol/L	34.30%	19.90%	3776
TS	7.50E-100	41(15)	37(17)	%	76.40%	17.10%	5378

* "Improved" refers to users who decreased higher than optimal levels by at least one zone; "Optimized" refers to users whose levels were in the optimal zone at draw 2 (see Methods for additional detail)
