# Supplementary material for: Improvements in blood and fitness tracker biomarkers in a longitudinal real-world cohort of digital health platform users
Source: PLOS Digit Health. 2026 Mar 24;5(3):e0001271. doi: 10.1371/journal.pdig.0001271 (PMC13012459; doi:10.1371/journal.pdig.0001271)
Supplement: S4 Table — (PDF) [file pdig.0001271.s004.pdf]

Table S2b. Users with lower than optimal baseline biomarker levels: baseline vs. follow-up

Biomarker	Mann-Whitney p	Draw 1 median(IQR)	Draw 2 median(IQR)	Unit	% users improved*	% users optimized*	Paired samples
RDW	0.34	11.4(0)	12.4(1.6)	%	78.90%	71.10%	38
ALT	2.40E-09	8(0)	10(3)	U/L	64.80%	64.80%	54
Fol	6.60E-31	4.941(0.3)	8.9665(6.4)	ng/mL	63.20%	62.70%	204
HCT	4.40E-22	37.5(1.9)	39.55(4.2)	%	61.40%	61.10%	352
Na	2.60E-176	136(1)	138(3)	mmol/L	65.30%	60.80%	1200
RBC_Mg	3.40E-67	3.8(0.2)	4.3(0.7)	mg/dL	59.20%	59.00%	424
MCHC	4.90E-142	31.4(0.7)	32.5(1.7)	g/dL	57.60%	57.50%	1374
MONOS	4.30E-47	10.25(170)	273(180)	cells/µL	57.40%	57.10%	310
Glu	1.80E-28	63.5315(1)	83(13)	mg/dL	78.40%	55.40%	231
EOS	3.90E-85	0(0.2)	60(100)	cells/µL	54.90%	54.60%	643
Cor	2.30E-20	4.7(0.2)	10.45(8.6)	µg/dL	68.60%	54.30%	140
NEUT	1.00E-87	1273.5(450)	1604(800)	cells/µL	53.50%	53.40%	820
MPV	0.88	9.2(0.8)	8.8(1.7)	fL	53.60%	52.00%	125
D	0.00E-01	26(7)	35(17)	ng/mL	57.10%	51.70%	5138
RBC	7.60E-10	4.08(0.16)	4.195(0.38)	$x10^6/\mu L$	49.90%	49.60%	349
Mg	4.20E-246	1.9(0.1)	2(0.2)	mg/dL	49.80%	49.40%	2274
PLT	5.00E-26	136(4)	152(32)	thousands/uL	48.90%	48.90%	235
LYMPHS	6.30E-44	600(800)	991(740)	cells/µL	48.80%	48.20%	361
TC	1.40E-48	120(5)	136(25)	mg/dL	49.30%	47.10%	446
TIBC	1.00E-38	240.224(9.8)	262(32)	ug/dL	47.20%	47.00%	362
CK	3.40E-08	40(5.5)	53(37)	U/L	49.60%	43.50%	131
WBC	3.50E-77	3.4(0.4)	3.7(1.1)	thousands/uL	45.30%	43.30%	1695
FE	2.80E-201	61(21)	92(46)	ug/dL	63.90%	35.60%	1574
Hb	1.20E-42	13.5(1.7)	13.8(1.6)	g/dL	33.80%	29.90%	5217
MCH	8.60E-20	26.2(1.2)	26.7(1.8)	pg	29.50%	29.50%	529
B12	1.90E-142	377(130)	437(200)	pg/mL	36.10%	27.90%	3423
MCV	1.00E-14	78.5(1.5)	81.1(5.2)	fL	27.50%	27.50%	298
TS	4.20E-172	21(6.2)	28(14)	%	50.50%	27.10%	2185
Tes	2.90E-62	415.25(120)	452(180)	ng/dL	30.00%	25.50%	3720
SHBG	2.80E-09	28(21)	32(22)	nmol/L	25.70%	22.80%	2316
HDL-c	3.40E-40	49(11)	51(15)	mg/dL	27.30%	20.20%	7977
FT	5.20E-49	6.62(2.1)	7.04(2.4)	ng/dL	24.90%	20.10%	4101
Fer	4.00E-21	29(22)	32(26)	ng/mL	21.80%	14.70%	4309
DHEAS	0.034	78(50)	79(58)	µg/dL	13.80%	12.00%	2140

* "Improved" refers to users who raised lower than optimal levels by at least one zone; "Optimized" refers to users whose levels were in the optimal zone at draw 2 (see Methods for additional detail)
