# Supplementary material for: Improvements in blood and fitness tracker biomarkers in a longitudinal real-world cohort of digital health platform users
Source: PLOS Digit Health. 2026 Mar 24;5(3):e0001271. doi: 10.1371/journal.pdig.0001271 (PMC13012459; doi:10.1371/journal.pdig.0001271)
Supplement: S5 Table — (PDF) [file pdig.0001271.s005.pdf]

Table S3. Correlation between polygenic traits and baseline blood biomarker

Polygenic risk score (PGS) trait	PGS identifier	Baseline blood biomarker phenotype	% phenotype explained by trait	P-value
mean platelet volume	GCST90078989	MPV	12.84%	<1.0E-294
total iron binding capacity	GCST011368	TIBC	10.86%	<1.0E-294
apoB levels	PGS001889	APOB	10.03%	1.24E-123
mean corpuscular hemoglobin	GCST90018964	MCH	9.50%	<1.0E-294
platelet count	PGS000186	PLT	8.29%	<1.0E-294
mean corpuscular volume	GCST90002338	MCV	6.95%	6.30E-293
low density lipoprotein cholesterol	PGS000310	LDL-c	5.88%	4.38E-250
high density lipoprotein cholesterol	GCST90239649	HDL-c	5.67%	1.21E-240
total cholesterol	PGS002286	TC	5.03%	4.53E-213
eosinophil count	GCST90002302	EOS	4.52%	1.40E-185
erythrocyte count	GCST90018971	RBC	3.58%	2.21E-149
red cell distribution width	GCST90002404	RDW	2.96%	5.96E-76
calcium levels	GCST90018951	Ca	2.94%	6.83E-75
triglyceride levels	PGS000312	Tg	2.71%	1.17E-114
white blood cell count	GCST90018978	WBC	2.71%	2.86E-70
neutrophil count	GCST90018968	NEUT	2.34%	2.18E-96
serum iron	GCST011367	FE	2.16%	1.44E-78
hsCRP levels	GCST009777	hsCRP	2.03%	4.44E-75
mean corpuscular hemoglobin concentration	GCST90018964	MCHC	1.92%	4.80E-80
serum gamma glutamyl transferase	GCST90018954	GGT	1.80%	4.95E-69
male testosterone levels	GCST90012113	Tes*	1.71%	1.02E-45
hemoglobin A1c levels	PGS002599	HgbA1c	1.67%	3.29E-68
hematocrit	GCST90002308	HCT	1.52%	2.94E-64
fasting blood glucose	PGS000838	Glu	1.51%	3.70E-64
serum 25-Hydroxyvitamin D levels	GCST010144	D	1.19%	2.65E-47
serum albumin levels	GCST90018945	Alb	0.91%	4.75E-38
aspartate aminotransferase levels	GCST90018944	AST	0.77%	7.51E-31
alanine transaminase levels	GCST90011898	ALT	0.67%	1.23E-27
magnesium levels	GCST000756	Mg	0.50%	8.76E-20
ferritin levels	GCST011369	Fer	0.31%	4.89E-13

*males only
