# Supplementary material for: Improvements in blood and fitness tracker biomarkers in a longitudinal real-world cohort of digital health platform users
Source: PLOS Digit Health. 2026 Mar 24;5(3):e0001271. doi: 10.1371/journal.pdig.0001271 (PMC13012459; doi:10.1371/journal.pdig.0001271)
Supplement: S6 Table — (PDF) [file pdig.0001271.s006.pdf]

Table S4. Correlation between polygenic traits and change in blood biomarker levels between baseline and follow-up test

Polygenic risk score trait	Delta blood biomarker phenotype	% phenotype explained by trait	p-value (T1vsT2)	p-value (T2vsT3)	p-value (T1vsT3)
ferritin	Fer	2.6 %	0.0031	0.78	0.012
high density lipoprotein cholesterol	HDL	0.6 %	0.35	0.8	0.34
apoB levels	APOB	0.4 %	0.58	0.95	0.68
creatine kinase	CK	0.3 %	0.24	0.69	0.67
low density lipoprotein cholesterol	LDL	0.2 %	0.26	0.048	0.029
total cholesterol	Chol	0.2 %	0.06	0.13	0.013
