# Supplementary material for: Improvements in blood and fitness tracker biomarkers in a longitudinal real-world cohort of digital health platform users
Source: PLOS Digit Health. 2026 Mar 24;5(3):e0001271. doi: 10.1371/journal.pdig.0001271 (PMC13012459; doi:10.1371/journal.pdig.0001271)
Supplement: S7 Table — (PDF) [file pdig.0001271.s007.pdf]

Table S5. Baseline and post-baseline personal fitness tracker data compared between users who improved high cholesterol levels compared to non-improved users

Fitness Tracker Biomarker*	P-value	Mean value: users who improve high cholesterol	Mean value: users who do not improve cholesterol
age	0.608	51 yrs	49 yrs
BMI at baseline	0.897	24 kg/m2	24 kg/m2
baseline cholesterol level	0.874	233 mg/dL	231 mg/dL
active calories at baseline	0.007	563 kcal	921 kcal
resting heart rate at baseline	0.306	59 bpm	57 bpm
vo2 max at baseline	0.085	41 mL/kg/min	47 mL/kg/min
daily step count at baseline	0.224	8587 steps	10143 steps
nightly sleep duration at baseline	0.322	6.6 hrs	7.0 hrs
nightly REM sleep % at baseline	0.064	22 %	19 %
nightly deep sleep % at baseline	0.323	8 %	11 %
change in active calories, baseline to post-baseline	0.130	54 kcal	-60 kcal
change in resting heart rate,, baseline to post-baseline	0.793	-0.4 bpm	-0.2 bpm
change in vo2 max, baseline to post-baseline	0.148	0.3 mL/kg/min	-1.1 mL/kg/min
change in daily step count, baseline to post-baseline	0.033	950 steps	-1060 steps
change in nightly sleep duration, baseline to post-baseline	0.094	0.2 hrs	-0.1 hrs
change in nightly REM sleep %, baseline to post-baseline	0.757	0 %	0 %
change in nightly deep sleep %, baseline to post-baseline	0.992	0 %	0 %

* Baseline fitness tracker data is collected in the 30 days prior to baseline blood draw. Post-baseline fitness tracker data is collected starting 5 days post-baseline blood draw, until follow-up blood draw.
