# Supplementary material for: Improvements in blood and fitness tracker biomarkers in a longitudinal real-world cohort of digital health platform users
Source: PLOS Digit Health. 2026 Mar 24;5(3):e0001271. doi: 10.1371/journal.pdig.0001271 (PMC13012459; doi:10.1371/journal.pdig.0001271)
Supplement: S8 Table — (PDF) [file pdig.0001271.s008.pdf]

Table S6a. Zone-transition outcomes by cohort

Action Plan targeting biomarker vs no Action Plan; biomarkers above optimal at baseline

Biomarker	Abbreviation	P-Value	Met (AP) n	Not met (AP) n	Met (noAP) n	Not met (noAP) n	Met (AP) %	Met (noAP) %	Risk differential
Glucose	Glu	1.0e-07	3565	4850	1214	2073	42.4%	36.9%	-5.4%
Low-Density Lipoprotein	LDL	4.0e-07	3001	9446	844	3314	24.1%	20.3%	-3.8%
Total Cholesterol	Chol	1.4e-06	3198	5594	981	2127	36.4%	31.6%	-4.8%
Creatine Kinase	CK	2.6e-04	679	809	319	524	45.6%	37.8%	-7.8%
Gamma-Glutamyl Transpeptidase	GGT	7.2e-04	1051	3335	316	1279	24.0%	19.8%	-4.2%
Triglycerides	Tg	2.1e-03	2925	3820	991	1499	43.4%	39.8%	-3.6%
Alanine Aminotransferase	ALT	2.5e-03	1959	3477	945	1943	36.0%	32.7%	-3.3%
Aspartate Aminotransferase	AST	2.5e-03	1524	1825	782	1116	45.5%	41.2%	-4.3%
Hemoglobin A1c	HgbA1c	3.2e-02	1764	4811	436	1357	26.8%	24.3%	-2.5%
C-Reactive Protein (high-sensitivity test)	hsCRP	3.1e-01	2165	4358	447	843	33.2%	34.7%	+1.5%
Cortisol	Cor	6.8e-	1003	1111	347	398	47.4%	46.6%	-0.9%
