# Supplementary material for: Improvements in blood and fitness tracker biomarkers in a longitudinal real-world cohort of digital health platform users
Source: PLOS Digit Health. 2026 Mar 24;5(3):e0001271. doi: 10.1371/journal.pdig.0001271 (PMC13012459; doi:10.1371/journal.pdig.0001271)
Supplement: S9 Table — (PDF) [file pdig.0001271.s009.pdf]

Table S6b. Zone-transition outcomes by cohort

Action Plan targeting biomarker vs no Action Plan; biomarkers below optimal at baseline

Biomarker	Abbreviation	P-Value	Met (AP) n	Not met (AP) n	Met (noAP) n	Not met (noAP) n	Met (AP) %	Met (noAP) %	Risk differential
Vitamin D	D	0.0e+00	3029	1594	760	741	65.5%	50.6%	-14.9%
Magnesium	Mg	0.0e+00	1198	930	277	367	56.3%	43.0%	-13.3%
Vitamin B12	B12	9.6e-06	951	1795	256	698	34.6%	26.8%	-7.8%
Hemoglobin	Hb	7.7e-05	660	1013	575	1170	39.5%	33.0%	-6.5%
Iron	FE	9.2e-02	33	27	208	270	55.0%	43.5%	-11.5%
Folate	Fol	9.8e-02	5	11	40	34	31.2%	54.1%	+22.8%
Red Blood Cell Magnesium	RBC_Mg	3.1e-01	276	155	83	57	64.0%	59.3%	-4.8%
High-Density Lipoprotein	HDL	4.6e-01	2015	5390	707	1964	27.2%	26.5%	-0.7%
Ferritin	Fer	5.5e-01	853	3135	270	946	21.4%	22.2%	+0.8%
