# Supplementary material for: Improvements in blood and fitness tracker biomarkers in a longitudinal real-world cohort of digital health platform users
Source: PLOS Digit Health. 2026 Mar 24;5(3):e0001271. doi: 10.1371/journal.pdig.0001271 (PMC13012459; doi:10.1371/journal.pdig.0001271)
Supplement: S10 Table — (PDF) [file pdig.0001271.s010.pdf]

Table S7. Quantification of Regression-to-the-Mean Effects by Biomarker

Biomarker	Baseline population mean	Test–retest correlation ($\rho_{1,2}$)	Observed change, Draw 1 → 2 (mean, 95% CI)	Expected change due to RTM	Observed change beyond RTM	RTM as % of observed change
Tes	407.77	0.91	120.7 (92.4, 149.0)	3.66	117.04	3.04%
HDL	59.48	0.93	3.0 (2.3, 3.6)	0.82	2.14	27.57%
D	37.11	0.57	12.4 (10.9, 14.0)	5.40	7.05	43.39%
HgbA1c	5.32	0.82	-0.1 (-0.1, -0.1)	-0.05	-0.06	45.79%
B12	650.82	0.77	140.4 (102.1, 178.7)	67.18	73.24	47.84%
LDL	114.20	0.80	-12.7 (-15.3, -10.0)	-8.24	-4.41	65.13%
Tg	96.79	0.74	-16.9 (-22.2, -11.5)	-11.50	-5.35	68.24%
FE	111.01	0.38	41.7 (33.3, 50.1)	32.91	8.79	78.91%
hsCRP	1.20	0.70	-0.5 (-0.9, -0.1)	-0.40	-0.10	80.87%
Glu	90.64	0.60	-4.0 (-5.0, -3.0)	-3.40	-0.63	84.3%
Fol	15.66	0.66	4.4 (3.5, 5.4)	3.96	0.47	89.33%
Cor	13.81	0.57	-2.7 (-3.3, -2.0)	-2.51	-0.18	93.2%
