# Supplementary material for: Improvements in blood and fitness tracker biomarkers in a longitudinal real-world cohort of digital health platform users
Source: PLOS Digit Health. 2026 Mar 24;5(3):e0001271. doi: 10.1371/journal.pdig.0001271 (PMC13012459; doi:10.1371/journal.pdig.0001271)
Supplement: S11 Table — (PDF) [file pdig.0001271.s011.pdf]

Table S8. Definitions and Sources of Biomarker “Optimal” Ranges
Optimal Zone commentary / explanation with source references

Marker	Optimal Zone Rationale
Albumin	Serum albumin levels in the upper clinical quartiles are strong predictors of longevity and reduced all-cause mortality. This optimal range is associated with lower risks of cardiovascular dysfunction and reflects a lack of systemic inflammation or oxidative stress. By applying the NHANES Macro subset methodology, this zone is defined using a healthy, non-diseased reference population to represent peak physiological resilience. This data-driven approach accounts for demographic variables to distinguish optimal function from standard clinical averages. Monitoring these levels allows for the early identification of metabolic shifts prior to the onset of chronic disease.<br><br>Citations<br><br>1. Schalk, B. W., et al. (2004). *Journal of Clinical Epidemiology*.<br><br>2. Centers for Disease Control and Prevention (CDC). National Center for Health Statistics (NCHS). National Health and Nutrition Examination Survey Data.
ALT	Alanine aminotransferase (ALT) is a critical enzyme for gluconeogenesis, and its blood concentration serves as a primary indicator of hepatocyte integrity and metabolic health. Research indicates a U-shaped relationship with mortality, where maintaining levels within a specific lower-mid range is associated with increased longevity and reduced cardiovascular risk. While elevated ALT can signal liver stress or metabolic syndrome, excessively low levels are often linked to frailty and decreased muscle mass in aging populations. Utilizing a healthy, non-diseased reference population from the NHANES dataset, we define an optimal zone that captures peak physiological resilience across different demographics. This data-driven approach allows for the identification of subtle shifts in liver and metabolic function before they progress toward chronic disease.<br><br>Citations<br><br>1. Lee, T. H., Kim, W. R., Benson, J. T., Therneau, T. M., & Melton, L. J., 3rd (2008). Serum aminotransferase activity and mortality risk in a United States community. Hepatology, 47(3), 880–887.<br><br>2. Centers for Disease Control and Prevention (CDC). National Center for Health Statistics (NCHS). National Health and Nutrition Examination Survey Data.

Marker	Optimal Zone Rationale
ApoB	Apolipoprotein B (ApoB) provides a direct measure of the total number of atherogenic lipoprotein particles, serving as a superior predictor of cardiovascular events and lifespan compared to traditional cholesterol markers. Longitudinal data and Mendelian randomization studies indicate that lower lifetime exposure to ApoB-containing particles is associated with increased longevity and a significantly reduced risk of coronary heart disease. Because each potentially harmful particle contains exactly one ApoB molecule, this marker allows for a more precise assessment of disease prevention potential than standard lipid panels. The optimal zone reflects levels associated with minimal risk of atherosclerotic progression and improved metabolic health. This target is highly personalized, as factors such as age, gender, and hormonal status (particularly menopausal transitions) significantly influence particle concentration.<br><br>Citations<br><br>1. Richardson, T. G., et al. (2021). Effects of apolipoprotein B on lifespan and risks of major diseases including type 2 diabetes: a mendelian randomisation analysis using outcomes in first-degree relatives. PLoS Medicine.<br><br>2. Ference, B. A., et al. (2019). Association of Genetic Variants Related to Combined Exposure to Lower Low-Density Lipoprotein Cholesterol and Lower Systolic Blood Pressure With Lifetime Risk of Cardiovascular Disease. JAMA.
AST	Aspartate aminotransferase (AST) is a key metabolic enzyme found in highly active tissues, including the liver, heart, and skeletal muscle. While elevated levels often signal cellular stress or tissue damage, research suggests that AST also serves as a broader marker of systemic health and longevity, with levels in the lower clinical range associated with decreased mortality risk. Importantly, the optimal zone for AST is highly sensitive to physical activity levels, as strenuous exercise, particularly resistance training, can cause transient elevations due to normal muscle repair rather than hepatic dysfunction. Because AST is found in greater net quantities in skeletal muscle than in the liver, personalized ranges must account for training status to distinguish between peak physiological adaptation and metabolic stress. Monitoring these levels within an activity-adjusted framework allows for a more precise assessment of an individual's internal resilience and recovery.<br><br>Citations<br><br>1. Lee, T. H., Kim, W. R., Benson, J. T., Therneau, T. M., & Melton, L. J., 3rd (2008). Serum aminotransferase activity and mortality risk in a United States community. Hepatology, 47(3), 880–887.<br><br>2. Pettersson, J., et al. (2008). Muscular exercise can cause highly pathological liver function tests in healthy men. British Journal of Clinical Pharmacology, 65(2), 253–259.

**Marker**	**Optimal Zone Rationale**
B12	Vitamin B12 is an essential cofactor for DNA synthesis, red blood cell formation, and the methylation of homocysteine, which is a marker linked to cardiovascular and neurological risk. Maintaining levels in the upper clinical range is associated with superior cognitive function and the prevention of megaloblastic anemia and peripheral neuropathy. The optimal zone is particularly critical for advanced athletes because endurance exercise can increase metabolic demand and elevate homocysteine levels, necessitating higher circulating B12 to maintain performance. While low levels often stem from inadequate intake or malabsorption, identifying marginal status early is key to preventing long term neurological decline. Consequently, personalized targets are adjusted for physical activity to support the increased metabolic turnover required for energy production and tissue repair.<br><br>Citations<br><br>1. Allen, L. H. (2009). How common is vitamin B-12 deficiency? The American Journal of Clinical Nutrition, 89(2), 693S-696S.<br><br>2. Herrmann, M., et al. (2003). Homocysteine increases during endurance exercise. Clinical Chemistry and Laboratory Medicine, 41(11), 1518-1524.
Basophills	Basophils are a specialized subset of leukocytes that play a critical role in the immune response to parasites and the mediation of allergic inflammation. Although they typically account for less than 1% of total white blood cells, elevated levels can serve as an early indicator of chronic inflammation, autoimmune activity, or hypersensitivity reactions. From a disease prevention perspective, maintaining basophils within a lower range is associated with a reduced risk of systemic inflammatory conditions and uremia. Conversely, exceptionally low levels may be linked to hyperthyroidism or an acute stress response. By prioritizing the lower end of the clinical range, this optimal zone reflects a state of immune homeostasis and minimal allergen sensitization.<br><br>Citations<br><br>1. Siracusa, M. C., et al. (2013). Basophils and inflammation. Journal of Allergy and Clinical Immunology, 132(4), 789–801.<br><br>2. Justiz Vaillant, A. A., & Stang, C. M. (2022). Lymphopenia. StatPearls Publishing.

**Marker**	**Optimal Zone Rationale**
Calcium	Serum calcium is an essential mineral required for nerve transmission, muscle contraction, and vascular health, with blood levels maintained within a narrow physiological range through tight hormonal regulation. Because the body will resorb calcium from bone tissue to maintain blood concentrations, levels in the lower clinical range may signal early mineral imbalances or vitamin D insufficiency. Conversely, elevations within the normal range can be associated with hyperparathyroidism or excessive bone turnover. The optimal zone is defined to reflect peak metabolic stability and bone mineral preservation, rather than just the absence of acute clinical symptoms. This range is further refined by age and gender to account for shifts in hormonal status and bone density throughout the lifespan.<br><br>Citations<br><br>1. Reid, I. R., et al. (2016). Calcium supplements and cardiovascular risk. Ageing Research Reviews.<br>2. Peacock, M. (2010). Calcium metabolism in health and disease. Clinical Journal of the American Society of Nephrology.
Cortisol	Cortisol is the primary glucocorticoid responsible for regulating the stress response, glucose metabolism, and immune function. Within the hierarchy of evidence, maintaining cortisol within a specific optimal zone is crucial for preventing long term muscle breakdown, loss of brain volume, and hypertension. While standard clinical ranges are broad, the optimal zone is narrowed based on the NHANES Macro subset regression to reflect healthy adrenal output that avoids the risks of both chronic elevation and adrenal insufficiency. This range is personalized by age and gender to account for natural shifts in the hypothalamic pituitary adrenal axis over the lifespan. Monitoring morning cortisol levels within this range allows for the early detection of HPA axis dysfunction before it progresses to clinical burnout or metabolic syndrome.<br><br>Citations<br><br>1. Kazlauskaite, R., et al. (2008). The serum cortisol test for screening of adrenocortical insufficiency in post-operative patients. Clinical Endocrinology.<br>2. Centers for Disease Control and Prevention (CDC). National Center for Health Statistics (NCHS). National Health and Nutrition Examination Survey Data.

Marker	Optimal Zone Rationale
Creatine Kinase	Creatine kinase is a vital enzyme for cellular energy homeostasis, particularly in high-demand tissues like skeletal muscle, the heart, and the brain. Elevated levels often serve as a biomarker for systemic inflammation or acute tissue damage, and are clinically associated with myocardial infarction and hypertension. Conversely, lower levels within the clinical range are frequently linked to decreased muscle mass, atrophy, or small stature, highlighting the importance of muscle as a longevity-promoting organ. Because CK is highly sensitive to physical activity, the optimal zone is personalized to distinguish between healthy muscle remodeling from exercise and pathological tissue breakdown. Monitoring this enzyme provides a window into both metabolic efficiency and structural resilience, ensuring that levels reflect peak muscular health rather than dysfunction.<br><br>Citations<br><br>1. Mougios, V. (2007). Reference intervals for serum creatine kinase in athletes. British Journal of Sports Medicine, 41(10), 674–678.<br><br>2. Miller, W. G., Chinchilli, V. M., Gruemer, H. D., & Nance, W. E. (1984). Sampling from a skewed population distribution as exemplified by estimation of the creatine kinase upper reference limit. Clinical Chemistry, 30(1), 18–23.
DHEAS (women)	DHEAS is a fundamental steroid precursor that plays a multi-faceted role in immunomodulation, neurosteroid activity, and the maintenance of bone and muscle integrity in women. DHEAS is a critical marker for longevity, as levels naturally decline after the third decade of life and lower concentrations are associated with immune senescence and cognitive impairment. Maintaining levels in the upper quartiles of age-adjusted ranges is linked to reduced vascular tension and a lower risk of non-alcoholic fatty liver disease. Utilizing the NHANES dataset, we establish an optimal floor based on a healthy, non-diseased population to ensure levels support active metabolic and hormonal health. This personalized approach allows for the preservation of physiological resilience by identifying accelerated hormonal decline before it manifests as age-related pathology.<br><br>Citations<br><br>1. Davison, S. L., Bell, R., Donath, S., Montalto, J. G., & Davis, S. R. (2005). Androgen levels in adult females: changes with age, menopause, and oophorectomy. Journal of Clinical Endocrinology & Metabolism, 90(7), 3847–3853.<br><br>2. Centers for Disease Control and Prevention (CDC). National Center for Health Statistics (NCHS). National Health and Nutrition Examination Survey Data.

Marker	Optimal Zone Rationale
Eosinophils	Eosinophils are specialized leukocytes essential for mediating the immune response to parasitic infections and maintaining systemic homeostasis. While they are central to type II immune reactions, such as asthma and allergies, chronically elevated levels are increasingly recognized as markers for autoimmune activity and metabolic syndrome. Maintaining a lower eosinophils level is associated with reduced systemic inflammation and a balanced immune profile. Conversely, exceptionally low counts can signal an acute bacterial infection or an overactive stress response involving high corticosteroid levels. By targeting this optimized range, practitioners can identify early shifts in immune balance before they manifest as chronic allergic or inflammatory pathologies.<br><br>Citations<br><br>1. Ramirez, G. A., et al. (2018). Eosinophils from Physiology to Disease: A Comprehensive Review. Biomed Res Int.
Estradiol	Estradiol is a primary regulator of reproductive health, bone density, and vascular integrity across the lifespan. For women, the optimal zone is defined by balancing systemic protection with long-term safety, as longitudinal data from the Nurses' Health Study II indicates that follicular and luteal levels in the highest quintiles are associated with a significantly increased risk of invasive breast cancer. Maintaining levels above a minimum threshold is also linked to protective effects against severe inflammatory disease and the preservation of bone mineral density. In men, maintaining a stable range is essential for preventing metabolic dysfunction while supporting libido and cognitive health. By monitoring these phase-specific targets, individuals can identify hormonal imbalances that impact both immediate physiological performance and long-term disease prevention.<br><br>Citations<br><br>1. Fortner, R. T., Eliassen, A. H., Spiegelman, D., Willett, W. C., Barbieri, R. L., & Hankinson, S. E. (2013). Premenopausal endogenous steroid hormones and breast cancer risk: results from the Nurses' Health Study II. Breast Cancer Research.<br><br>2. Eliassen, A. H., Missmer, S. A., Tworoger, S. S., Spiegelman, D., Barbieri, R. L., Dowsett, M., & Hankinson, S. E. (2006). Endogenous steroid hormone concentrations and risk of breast cancer among premenopausal women. Journal of the National Cancer Institute.

Marker	Optimal Zone Rationale
Ferritin	Ferritin serves as the primary storage protein for iron, and its circulating levels are a critical indicator of total body iron reserves and systemic health. Within the hierarchy of evidence, maintaining ferritin in an optimal range is vital for preventing iron-deficiency anemia while avoiding the oxidative stress associated with iron overload. For advanced athletes, the optimal range is shifted higher to support increased red blood cell turnover and the metabolic demands of high-level cardiovascular fitness. Conversely, elevated ferritin can act as an acute-phase reactant signaling chronic inflammation or insulin resistance, particularly in postmenopausal populations. By personalizing these targets based on age, sex, and activity levels, practitioners can distinguish between healthy storage and subclinical inflammatory states.<br><br>Citations<br><br>1. De Matos, L. D. N. J., et al. (2011). Cardiovascular risk and clinical factors in athletes: 10 years of evaluation. Medicine & Science in Sports & Exercise.<br>2. Mainous, A. G., 3rd, & Diaz, V. A. (2009). Relation of serum ferritin level to cardiovascular fitness among young men. The American Journal of Cardiology.<br>3. Knovich, M. A., Storey, J. A., Coffman, L. G., Torti, S. V., & Torti, F. M. (2009). Ferritin for the clinician. Blood Reviews, 23(3), 95–104.
Folate	Folate is a critical B vitamin required for the development of red and white blood cells, as well as the regulation of methionine and homocysteine metabolism. Maintaining folate within this optimal zone is essential for managing homocysteine levels, particularly for those engaging in high intensity athletic activity or endurance exercise, which has been shown to increase homocysteine concentrations. Within the hierarchy of evidence for longevity, adequate folate supports DNA synthesis and cellular repair while reducing cardiovascular and cancer risk. Furthermore, emerging research highlights novel interactions between folate and lipid metabolism, suggesting its role extends to broader metabolic health. Monitoring folate levels is also vital because elevated serum concentrations can sometimes signal an underlying vitamin B12 insufficiency, as B12 is a mandatory cofactor for proper folate metabolism.<br><br>Citations<br><br>1. Herrmann, M., et al. (2003). Homocysteine increases during endurance exercise. Clinical Chemistry and Laboratory Medicine.<br>2. da Silva, R. P., et al. (2014). Novel insights on interactions between folate and lipid metabolism. BioFactors.

Marker	Optimal Zone Rationale
Free Testosterone (men)	Free testosterone represents the unbound, physiologically active fraction of the hormone that is readily available to tissues. Because it is not bound to sex hormone-binding globulin (SHBG), its measurement provides a more accurate assessment of androgen status than total testosterone alone, especially when SHBG levels are elevated. Maintaining free testosterone within the optimal range is critical for preserving muscle mass, regulating blood pressure, and supporting metabolic health. Research indicates that testosterone deficiency is linked to an increased risk of mortality, while optimizing these levels has been shown to improve survival rates in men with metabolic conditions like type 2 diabetes. Low levels are often associated with late-onset hypogonadism, which can manifest as anemia, increased inflammation, and reduced physical resilience.<br><br>Citations<br><br>1. Zarotsky, V., et al. (2014). Systematic literature review of the risk factors, comorbidities, and consequences of hypogonadism in men. Andrology.<br><br>2. Muraleedharan, V., et al. (2013). Testosterone deficiency is associated with increased risk of mortality and testosterone replacement improves survival in men with type 2 diabetes. European Journal of Endocrinology
GGT	Gamma-glutamyltransferase (GGT) is a vital enzyme for glutathione metabolism and a sensitive biomarker for oxidative stress and metabolic health. GGT serves as a "metabolic clue" for cardiovascular disease, insulin resistance, and all-cause mortality. Higher GGT levels are progressively associated with increased coronary artery calcification, insulin resistance, and even certain cancers. Conversely, lower levels are associated with better cardiovascular fitness and lower fasting glucose. Maintaining GGT within this optimal zone reflects efficient antioxidant status, liver health, metabolic syndrome, and cardiovascular risk.<br><br>Citations<br><br>1. Yousefzadeh, Gholamreza et al. “Role of gamma-glutamyl transferase (GGT) in diagnosis of impaired glucose tolerance and metabolic syndrome: a prospective cohort research from the Kerman Coronary Artery Disease Risk Study (KERCADRS).” Diabetes & metabolic syndrome.<br><br>2. Centers for Disease Control and Prevention (CDC). National Center for Health Statistics (NCHS). National Health and Nutrition Examination Survey Data.

**Marker**	**Optimal Zone Rationale**
Glucose	Glucose is the primary fuel source for cellular energy, yet its regulation must be maintained within a narrow range to prevent systemic damage. Elevated levels serve as a hallmark of metabolic dysfunction and are associated with visceral obesity, hypertension, and cardiovascular disease. Longitudinal data from the Framingham Heart Study indicates that while blood glucose tends to increase with age, the level corresponding to the lowest mortality risk also shifts, reflecting an age-related decline in stress resistance. Conversely, low levels define clinical hypoglycemia, which can cause neuroglycopenic symptoms and autonomic dysfunction.<br><br>**Citations**<br><br>1. Yashin, A. I., et al. (2009). Maintaining physiological state for exceptional survival: What is the normal level of blood glucose and does it change with age? Mechanisms of Ageing and Development.<br><br>2. Field, J. B. (1989). Hypoglycemia. Definition, clinical presentations, classification, and laboratory tests. Endocrinology and Metabolism Clinics of North America.
HbA1c	HbA1c measures the percentage of hemoglobin bound to glucose over time, serving as a critical indicator of long-term glycemic control. HbA1c has been shown to provide superior prognostic value compared to fasting glucose for predicting cardiovascular disease and all-cause mortality in literature. Research in nondiabetic adults shows that as HbA1c levels rise, the risk for coronary heart disease and diagnosed diabetes increases significantly. Conversely, a J-shaped association with mortality suggests that while elevated levels indicate chronic hyperglycemia, exceptionally low levels may reflect chronic hypoglycemia or increased red blood cell turnover. Maintaining levels within this optimal zone ensures efficient tissue oxygenation and minimizes the risk of vascular damage associated with glycosylation.<br><br>Citations<br><br>1. Centers for Disease Control and Prevention (CDC). National Center for Health Statistics (NCHS). National Health and Nutrition Examination Survey Data.<br><br>2. Selvin, E., et al. (2010). Glycated hemoglobin, diabetes, and cardiovascular risk in nondiabetic adults. New England Journal of Medicine.

Marker	Optimal Zone Rationale
HDL	High-density lipoprotein (HDL) serves as a scavenger, returning cholesterol from the periphery to the liver for processing. Research indicates that while low HDL is a well-known risk factor for cardiovascular disease and metabolic syndrome, extreme high concentrations are paradoxically associated with high all-cause mortality. Studies show a U-shaped association between HDL concentrations and mortality in both men and women, with the lowest risk found at moderate levels. Furthermore, current research highlights a paradigm shift focusing on HDL quality and functionality rather than just quantity, as modified HDL can become pathogenic in the presence of inflammation or toxins. Maintaining levels within this optimal zone is essential to ensure HDL continues to provide neuroprotective and cardiovascular benefits.<br><br>Citations<br><br>1. Madsen, C. M., et al. (2017). Extreme high high-density lipoprotein cholesterol is paradoxically associated with high mortality in men and women: two prospective cohort studies. European Heart Journal.<br>2. Cho, Kyung-Hyun. (2022). The Current Status of Research on High-Density Lipoproteins (HDL): A Paradigm Shift from HDL Quantity to HDL Quality and HDL Functionality. International Journal of Molecular Sciences.
Hematocrit	Hematocrit measures the percentage of total blood volume composed of red blood cells and serves as a primary determinant of blood viscosity, flow, and platelet adhesion. Research indicates that maintaining levels within a range more precise than clinically normal is essential for ensuring efficient oxygen delivery to tissues without compromising circulation. Studies show that elevated hematocrit can be associated with insulin resistance, dehydration, or testosterone therapy, potentially increasing cardiovascular strain. Conversely, lower levels are frequently linked to nutrient insufficiencies, chronic disease, or anemia, which may significantly impact energy levels and physiological resilience.<br><br>Citations<br><br>1. Jin, Y. Z., et al. (2014). Relationship Between Hematocrit Level and Cardiovascular Risk Factors in a Community-Based Population. Journal of Clinical Laboratory Analysis.<br>2. Taylor, C. S., et al. (2023). Hematocrit. StatPearls [Internet].

<table>
<tr><th>Marker</th><th>Optimal Zone Rationale</th></tr>
<tr><td>Hemoglobin</td><td>Hemoglobin is the iron-containing globular protein in red blood cells responsible for systemic oxygen transport. Research indicates that maintaining levels within a range more precise than clinically normal is vital, as a reduction can signal anemia or nutrient deficiencies that impair tissue oxygenation. Conversely, elevated levels may be associated with dehydration, chronic disease, or certain hormonal therapies. While physical training provides cardioprotective benefits, it does not eliminate the prevalence of cardiovascular risk factors that may influence hematological profiles.

Citations

1. De Matos, L. D. N. J., et al. (2011). Cardiovascular risk and clinical factors in athletes: 10 years of evaluation. Medicine & Science in Sports & Exercise.

2. Kuipers, H., et al. (2007). Hemoglobin levels and athletic performance in elite speed skaters during the olympic season 2006. Clinical Journal of Sport Medicine.</td></tr>
<tr><td>hsCRP</td><td>High-sensitivity CRP (hs-CRP) is an acute-phase reactant produced by the liver that serves as a highly sensitive marker for systemic inflammation. Studies show that even moderate elevations are associated with the progression of atherosclerosis, non-alcoholic fatty liver disease (NAFLD), and metabolic syndrome. Furthermore, elevated hs-CRP and low HDL cholesterol have been found to jointly contribute to the prediction of all-cause, cancer, and cardiovascular mortality. In older populations, hs-CRP and interleukin-6 (IL-6) serve as powerful predictors of mortality, often outperforming conventional risk scores.

Citations.

1. Yu, E., et al. (2018). Inflammatory Biomarkers and Risk of Atherosclerotic Cardiovascular Disease. Open Medicine.

2. Kim, K. I., et al. (2012). CRP level and HDL cholesterol concentration jointly predict mortality in a Korean population. The American Journal of Medicine.

3. Störk, S., et al. (2006). Prediction of mortality risk in the elderly. The American Journal of Medicine.</td></tr>
</table>

**Marker**	**Optimal Zone Rationale**
Insulin	Insulin is a vital hormone that facilitates the cellular uptake of glucose and nutrients for metabolism. Research indicates that elevated fasting insulin predicts the future incidence of metabolic syndrome. Furthermore, research highlights significant associations between fasting insulin levels and overall mortality risk. High fasting insulin is a hallmark of insulin resistance and metabolic dysfunction, often preceding the onset of hyperglycemia by several years.<br><br>Citations<br><br>1. Wiebe, N., et al. (2022). Associations of body mass index, fasting insulin, and inflammation with mortality: a prospective cohort study. International Journal of Obesity.<br>2. Sung, K. C., et al. (2011). Elevated fasting insulin predicts the future incidence of metabolic syndrome: a 5-year follow-up study. Cardiovascular Diabetology.
Iron	Iron is a fundamental mineral primarily utilized within hemoglobin to facilitate oxygen transport to tissues throughout the body. Studies show that maintaining serum iron within a range more precise than clinically normal is essential for preventing the cognitive and behavioral dysfunction associated with subclinical deficits. Research indicates that while low levels often stem from insufficient absorption or blood loss, elevated levels can signal oxidative stress, lead toxicity, or liver disease.<br><br>Citations<br><br>1. Centers for Disease Control and Prevention (CDC). National Center for Health Statistics (NCHS). National Health and Nutrition Examination Survey Data.
LDL	Low-density lipoproteins (LDL) transport cholesterol remaining after the removal of triglycerides from very low-density lipoproteins. Research indicates that maintaining LDL within a range more precise than clinically normal is significant, as studies show that low levels are associated with an increased risk of all-cause mortality and intracerebral hemorrhage. Conversely, elevated LDL levels, particularly when the particles are small, dense, and oxidized, contribute to the development of atherosclerosis and cardiovascular disease. Factors such as genetics and vitamin D status can further influence circulating levels and particle atherogenicity.<br><br>Citations<br><br>1. Centers for Disease Control and Prevention (CDC). National Center for Health Statistics (NCHS). National Health and Nutrition Examination Survey Data.<br>2. Penson, P. E., et al. (2018). Low-density lipoprotein-cholesterol and all-cause mortality.

Marker	Optimal Zone Rationale
Lymphocytes	Lymphocytes are white blood cells that protect against microbial infection and help regulate inflammation. Studies show that decreased lymphocyte levels are associated with immune compromise, more severe infection, and inflammation. Research also indicates that low lymphocytes are linked to all-cause mortality and cardiovascular disease. Conversely, elevated levels are associated with chronic infection, leukemia, lymphoma, metabolic syndrome, type 2 diabetes, and steroid use.<br><br>Citations<br><br>1. Zidar, D. A., et al. (2019). Association of Lymphopenia With Risk of Mortality Among Adults in the US General Population
Magnesium	Magnesium is a critically important mineral with antioxidant and anti-inflammatory properties, serving as a necessary cofactor for more than 600 metabolic enzymes. It is essential for cardiovascular health, neurological function, bone integrity, and glucose regulation. Studies show that low magnesium is associated with hypertension, cardiac arrhythmias, cardiovascular disease (CVD), and osteoporosis. Research also indicates that hypomagnesemia is linked to glucose dysregulation, systemic inflammation, headaches, depression, and cognitive impairment. Furthermore, insufficient levels can contribute to sleep disturbances, muscle cramps, and the formation of kidney stones. While hypermagnesemia is uncommon, it can occur with excessive intake or significantly reduced kidney excretion.<br><br>Citations<br><br>1. Costello, R. B., et al. (2016). Perspective: The Case for an Evidence-Based Reference Interval for Serum Magnesium: The Time Has Come. Advances in Nutrition.<br><br>2. Topf, J. M., & Murray, P. T. (2003). Hypomagnesemia and hypermagnesemia. Reviews in Endocrine and Metabolic Disorders.
MCH	Mean corpuscular hemoglobin (MCH) reflects the average amount of hemoglobin found in an individual red blood cell. Since hemoglobin is the primary vehicle for oxygen transport, MCH levels are inherently linked to red blood cell size; larger, macrocytic cells typically contain more hemoglobin, while smaller, microcytic cells contain less. Studies show that low MCH is associated with microcytic, hypochromic anemia and thalassemia. Specifically, research indicates that MCH is a sensitive screening tool for alpha-thalassemia-1 and beta-thalassemia traits. Conversely, elevated MCH levels are associated with macrocytic anemia and hemochromatosis. Evaluation of this marker is a key component in categorizing various anemias and red blood cell-related disorders.<br><br>Citations<br><br>1. Pranpanus, S., et al. (2009). Sensitivity and specificity of mean corpuscular hemoglobin (MCH): for screening alpha-thalassemia-1 trait and beta-thalassemia trait. J Med Assoc Thai.

**Marker**	**Optimal Zone Rationale**
MCHC	MCHC measures the average concentration of hemoglobin within a red blood cell. Studies show low levels are associated with hypochromic cells, iron-deficiency anemia, and thalassemia. Research also links low MCHC to systemic inflammation, poorer post-MI outcomes, and depressive symptoms, particularly in women. Conversely, high MCHC can indicate hemolysis, spherocytosis, or intracellular dehydration, though these are often associated with laboratory interference or specific medications.<br><br>Citations<br><br>1. Huang, Y. L., & Hu, Z. D. (2016). Lower mean corpuscular hemoglobin concentration is associated with poorer outcomes in intensive care unit admitted patients with acute myocardial infarction. Annals of Translational Medicine.<br>2. Lee, J. M., et al. (2017). Association between Mean Corpuscular Hemoglobin Concentration and Future Depressive Symptoms in Women. The Tohoku Journal of Experimental Medicine.
MCV	MCV measures the average size of red blood cells (RBCs) and is used to classify anemias. High MCV (macrocytosis) is associated with B12 or folate deficiency, liver disease, and hypothyroidism. Low MCV (microcytosis) is linked to iron or B6 deficiency, thalassemia, and lead poisoning. Because very large and very small cells can "average out" to a normal value, MCV should be evaluated with RDW. In chronic diseases like diabetes or malignancy, MCV may appear low or normal.<br><br>Citations<br><br>1. Cappellini, M. D., & Motta, I. (2015). Anemia in Clinical Practice-Definition and Classification: Does Hemoglobin Change With Aging? Seminars in Hematology.
Monocytes	Monocytes are white blood cells that play an active role in immune defense but can also become pathogenic, potentially damaging tissues and even supporting tumor growth. They differentiate into macrophages but can also be converted into osteoclasts and microglial cells. Elevations in monocytes are associated with low-grade inflammation seen with CVD, metabolic syndrome, diabetes, and other inflammatory disorders. Monocytes are also associated with infections and autoimmune disorders. Low levels may be seen with certain malignancies, immune disorders, and corticosteroid use.<br><br>Citations:<br><br>1. Seidler, S., et al. (2010). Age-dependent alterations of monocyte subsets and monocyte-related chemokine pathways in healthy adults. BMC Immunology.

**Marker**	**Optimal Zone Rationale**
MPV	MPV reflects the average size and production rate of platelets in the bone marrow. An increasing MPV indicates accelerated production, resulting in larger, more reactive platelets that increase the risk of complications. High MPV is associated with thrombocytopenia, B12 or folate deficiency, diabetes, metabolic syndrome, and chronic low-grade inflammation. It also serves as a marker for increased risk of thrombosis, myocardial infarction, and vascular risk. Conversely, low MPV is linked to aplastic anemia and high-grade inflammatory conditions, such as active rheumatoid arthritis.<br><br>Citations:<br><br>1. Gasparyan, A. Y., et al. (2011). Mean platelet volume: a link between thrombosis and inflammation? Current Pharmaceutical Design.
Neutrophils	Neutrophils are the most abundant white blood cells and serve as the primary defense against pathogens. They neutralize microorganisms by producing potent compounds and engulfing bacteria through phagocytosis. Elevated levels appear early in inflammation and are associated with infection, cardiovascular disease, and metabolic syndrome. Increases are also noted during trauma or emotional distress. Conversely, low levels may indicate bone marrow failure, radiation exposure, viral infections, or immune suppression.<br><br>Citations:<br><br>1. Rosales, C. (2018). Neutrophil: A Cell with Many Roles in Inflammation or Several Cell Types? Frontiers in Physiology.
Platelets	Platelets facilitate clotting and maintain blood volume during vascular injury. Both high and low levels are linked to increased all-cause mortality, making a balance of mature platelets vital to homeostasis. Low platelet counts are seen with bone marrow failure, hemorrhage, and certain anemias, and are associated with increasing fibrosis in NAFLD. Conversely, high counts are associated with iron-deficiency anemia, malignancy, and systemic inflammation. Research also indicates that platelet counts undergo significant changes during the aging process, which may influence clinical interpretation.<br><br>Citations<br><br>1. Balduini, C. L., & Noris, P. (2014). Platelet count and aging. Haematologica.

Marker	Optimal Zone Rationale
Potassium	Potassium is an essential electrolyte vital for muscle contractions, nerve transmission, and heart rate regulation. The kidneys maintain serum levels within a tight range, as extreme fluctuations can be fatal. Research indicates that both low and high potassium concentrations are associated with a higher risk of cardiovascular events and all-cause mortality in community-living individuals. Specifically, low levels are linked to hypertension, heart failure, and ventricular fibrillation, while high levels are often associated with renal failure and metabolic acidosis. Potassium status can also be compromised by stress, dehydration, and certain medications like diuretics.<br><br>Citations<br><br>1. Hughes-Austin, J. M., et al. (2017). The Relation of Serum Potassium Concentration with Cardiovascular Events and Mortality in Community-Living Individuals. Clinical Journal of the American Society of Nephrology.
Progesterone	Progesterone supports fertility, pregnancy, and cardiovascular, neurological, and bone health. In women, low levels are linked to infertility, miscarriage, PCOS, and premenstrual fatigue. Research also suggests that high levels of endogenous progesterone in premenopausal women may be associated with an increased risk of subsequent breast cancer. In men, low progesterone may compromise immunity, metabolism, and cardiovascular health, while high levels are associated with prediabetes and type 2 diabetes.<br><br>Citations<br><br>1. Micheli, A., et al. (2004). Endogenous sex hormones and subsequent breast cancer in premenopausal women. International Journal of Cancer.<br><br>2. Unfer, V., et al. (2005). Different routes of progesterone administration and polycystic ovary syndrome: a review of the literature. Gynecological Endocrinology.
RBC Magnesium	RBC magnesium reflects intracellular status, offering a better view of long-term stores than serum levels. Research indicates that low magnesium is associated with cardiovascular disease, vascular calcification, and endothelial dysfunction. Studies show that insufficiency contributes to poor glycemic control, cognitive impairment, and increased risks for hip fractures and Alzheimer's. Low levels are also linked to systemic inflammation, sleep disturbances, and depression. While high levels are atypical, they usually result from excessive supplementation or impaired kidney excretion.<br><br>Citations<br><br>1. Witkowski, M., et al. (2011). Methods of assessment of magnesium status in humans: a systematic review. Magnesium Research.

Marker	Optimal Zone Rationale
RDW	RDW measures the variation in red blood cell size. Studies show that high RDW is associated with nutritional deficiencies in iron, B12, or folate, as well as hemolytic and posthemorrhagic anemias. Research also indicates that elevated RDW is linked to chronic liver disease, metabolic syndrome, and increased inflammatory biomarkers such as CRP and fibrinogen. Furthermore, increased RDW is associated with higher coronary artery calcification scores, heart failure, and diabetes. High levels may also predict poorer cancer prognosis and increased risk for fatal and non-fatal cardiovascular events. Low RDW indicates a healthy uniformity in red blood cell width.<br><br>Citations:<br>1. Fava, C., et al. (2019). The role of red blood cell distribution width (RDW) in cardiovascular risk assessment: useful or hype? Annals of Translational Medicine.<br>2. Xanthopoulos, A., et al. (2017). Red blood cell distribution width as a prognostic marker in patients with heart failure and diabetes mellitus. Cardiovascular Diabetology.
Red Blood Cells	Red blood cells (RBCs) are essential for delivering oxygen to tissues and returning carbon dioxide to the lungs. Low RBC counts, or anemia, can stem from bone marrow failure, nutrient deficiencies, renal disease, or chronic illness, and are associated with increased cardiovascular risk and mortality. Conversely, a high RBC count (erythrocytosis) thickens the blood, which raises the risk of blood clots, strokes, and heart attacks. This elevation often results from dehydration, lung disease, or polycythemia vera.<br><br>Citations:<br>1. Madjid, M., & Fatemi, O. (2013). Components of the Complete Blood Count as Risk Predictors for Coronary Heart Disease. Tex Heart Inst.
SHBG	Sex hormone-binding globulin (SHBG) is a liver-derived protein that binds and transports sex hormones, regulating their bioavailability to tissues. Low levels are a significant marker for cardiometabolic dysfunction, often associated with insulin resistance, type 2 diabetes, PCOS, and chronic inflammation. Conversely, elevated levels can be seen in cases of hyperthyroidism or malnutrition and may lead to symptomatic hypogonadism by excessively binding active hormones. Maintaining SHBG within an optimal range is essential for hormonal balance and reflects a lack of underlying metabolic stress.<br><br>Citations:<br>1. Wang, Y. (2021). Definition, Prevalence, and Risk Factors of Low Sex Hormone-Binding Globulin in US Adults. The Journal of Clinical Endocrinology & Metabolism.<br>2. Gyawali, P., et al. (2019). Higher Serum Sex Hormone-Binding Globulin Levels Are Associated With Incident Cardiovascular Disease in Men. The Journal of Clinical Endocrinology & Metabolism.

**Marker**	**Optimal Zone Rationale**
Sodium	Sodium is an essential mineral crucial for nerve transmission, cell membrane transport, acid-base balance, and osmotic pressure. Its levels are regulated by a complex interplay of hormones, enzymes, and kidney function to maintain blood pressure and fluid equilibrium. Low levels (hyponatremia) are often associated with hyperglycemia, overhydration, or the use of specific medications like diuretics and NSAIDs, and may signal conditions such as congestive heart failure or adrenal insufficiency. Conversely, high levels (hypernatremia) typically reflect dehydration, excessive intake, or hormonal imbalances like hyperaldosteronism and Cushing syndrome.<br><br>Citations:<br>1. Dmitrieva, N. I., et al. (2023). Middle-age high normal serum sodium as a risk factor for accelerated biological aging, chronic diseases, and premature mortality. eBioMedicine.<br>2. Liamis, G., et al. (2013). Diabetes mellitus and electrolyte disorders. World Journal of Diabetes.
Testosterone	Testosterone is the primary androgenic hormone essential for sexual health, muscle integrity, and red blood cell production in both men and women. In men, maintaining levels within an optimal upper-tier range supports cardiometabolic resilience, whereas low levels are associated with visceral adiposity, andropause, and chronic opioid or steroid use. In women, physiological levels of testosterone are neuroprotective and support cognitive health; however, deficiencies often follow menopause or oophorectomy, leading to loss of libido and memory decline. Conversely, elevated levels in women are frequent indicators of PCOS or metabolic dysfunction, which can increase the risk of type 2 diabetes and atrial fibrillation.<br><br>Citations<br>1. Davis, S. R., et al. (2015). Testosterone in women—the clinical significance. The Lancet Diabetes & Endocrinology.<br>2. Hogervorst, E., et al. (2010). Low free testosterone is an independent predictor of cognitive impairment in older men. International Journal of Geriatric Psychiatry.

Marker	Optimal Zone Rationale
TIBC	Total Iron Binding Capacity (TIBC) measures the blood's total capacity to bind and transport iron, primarily reflecting the concentration of transferrin. In cases of iron deficiency, TIBC levels typically rise as the body increases transferrin production to maximize the transport of limited iron stores. Conversely, low TIBC levels are often observed in states of iron overload, where transport proteins decrease, or in chronic inflammatory conditions, liver cirrhosis, and hypoproteinemia. Monitoring TIBC is particularly important for active individuals, as endurance and weight-training exercise can significantly influence iron turnover and transport demands.<br><br>Citations<br><br>1. Woolf, K., et al. (2009). Iron status in highly active and sedentary young women. International Journal of Sport Nutrition and Exercise Metabolism.<br><br>2. Deruisseau, K. C., et al. (2004). Iron status of young males and females performing weight-training exercise. Medicine & Science in Sports & Exercise.
Total Cholesterol	Total cholesterol is a vital lipid essential for synthesizing cell membranes, steroid hormones, Vitamin D, and bile acids. It plays a critical role in the brain, particularly in forming the protective myelin sheath around nerve cells. While high levels are often associated with genetic factors, hypothyroidism, and an increased risk of cardiovascular disease (CVD) through atherosclerotic plaque formation, excessively low levels are also concerning. Suboptimal cholesterol is linked to malnutrition, depression, neurodegenerative diseases like Parkinson’s, and disrupted hormone metabolism. Maintaining cholesterol within an optimal window ensures sufficient raw materials for cellular function while minimizing the risk of oxidative damage and vascular inflammation.<br><br>Citations<br><br>1. Zubeldia-Brenner, L., et al. (2016). Developmental and Functional Effects of Steroid Hormones on the Neuroendocrine System. Frontiers in Neuroendocrinology.<br><br>2. Jin, S. S., et al. (2019). Plasma Cholesterol Levels and Risk of Parkinson’s Disease: A Meta-analysis. Clinical Psychopharmacology and Neuroscience.

Marker	Optimal Zone Rationale
Transferrin Saturation	Transferrin saturation (TSAT) represents the percentage of iron-transporting proteins currently occupied by iron, serving as a dynamic indicator of iron availability for red blood cell production. Maintaining saturation within a stable window ensures that tissues receive adequate iron for energy metabolism without inducing the oxidative stress associated with "free" or unbound iron. Suboptimal levels are highly sensitive markers for iron deficiency, insulin resistance, and chronic inflammation, which can impair the body's ability to utilize its iron stores. Conversely, levels at the higher end of the spectrum can signal iron overload conditions or various megaloblastic anemias, increasing the risk of vascular damage and metabolic dysfunction.<br><br>Citations:<br><br>1. Stack, A. G., et al. (2014). Transferrin saturation and risk of total and cardiovascular mortality in the general population. QJM: An International Journal of Medicine.<br>2. Ellervik, C., et al. (2011). Elevated transferrin saturation and risk of diabetes: three population-based studies. Diabetes Care.
Triglycerides	Triglycerides are the primary storage form of energy in the body, but their circulating levels serve as a key indicator of metabolic flexibility. While clinical ranges focus on the prevention of acute conditions like pancreatitis, an optimized level reflects efficient fat oxidation and low visceral adiposity. Elevated levels are frequently associated with insulin resistance, fatty liver, and an increased risk of cardiovascular disease, especially when the triglyceride-to-HDL ratio is high. Conversely, very low levels are less common but may signal malnutrition or malabsorption, though they often simply reflect a highly active lifestyle and high metabolic efficiency.<br><br>Citations<br><br>1. Nordestgaard, B. G. (2016). Triglyceride-Rich Lipoproteins and Ischemic Heart Disease. Circulation Research.<br>2. Talayero, B. G., & Sacks, F. M. (2011). The Role of Triglycerides in Atherosclerosis. Current Cardiology Reports.

Marker	Optimal Zone Rationale
TSH	Thyroid-stimulating hormone (TSH) is produced by the pituitary gland and acts as the master regulator of thyroid hormone production and basal metabolic rate. Maintaining levels within a stable window supports efficient energy production, temperature regulation, and lipid metabolism. High TSH indicates an underactive thyroid (hypothyroidism), which can lead to fatigue, weight gain, and an increased risk of metabolic syndrome. Conversely, low TSH signals an overactive state (hyperthyroidism) or pituitary hypofunction, which can cause heart palpitations and bone loss.<br><br>Citations<br><br>1. Oh, J. Y., et al. (2013). Elevated thyroid stimulating hormone levels are associated with metabolic syndrome in euthyroid young women. Korean Journal of Internal Medicine.<br><br>2. Feldt-Rasmussen, U., & Klose, M. (2020). Clinical Strategies in the Testing of Thyroid Function. Endotext.
Vitamin D	Vitamin D is a critical hormone precursor that is converted into its active form, calcitriol, to regulate calcium absorption and immune function. Maintaining levels within a stable physiological window supports bone density, muscle recovery, and cardiovascular resilience. Insufficiency is widely associated with bone disease, systemic inflammation, and a higher risk of all-cause mortality. Conversely, excessive levels (typically from over-supplementation) can lead to calcium dysregulation, dehydration, and a paradoxically increased mortality risk, following a "reverse J-shaped" association. Monitoring levels ensures the body has the necessary precursors for long-term skeletal health and metabolic homeostasis.<br><br>Citations<br><br>1. Durup, D., et al. (2012). A reverse J-shaped association of all-cause mortality with serum 25-hydroxyvitamin D in general practice: the CopD study. The Journal of Clinical Endocrinology & Metabolism.<br><br>2. Pilz, S., et al. (2016). Vitamin D and Mortality. Anticancer Research.

<table>
<tr><th>Marker</th><th>Optimal Zone Rationale</th></tr>
<tr><td>WBC</td><td>White blood cells (WBCs) are the primary cellular defense of the immune system, responsible for neutralizing pathogens and mediating the inflammatory response. An optimized WBC count reflects an immune system that is vigilant but not chronically over-activated by systemic stressors. Low counts (leukopenia) can leave the body vulnerable to infection and may signal bone marrow fatigue or autoimmune activity. Conversely, counts trending toward the high-normal spectrum serve as a significant marker for chronic low-grade inflammation and increased cardiovascular risk. Monitoring these levels provides a window into systemic immune homeostasis and the presence of underlying metabolic stress.

Citations:

1. Madjid, M., & Fatemi, O. (2013). Components of the Complete Blood Count as Risk Predictors for Coronary Heart Disease: In-Depth Review and Update. Texas Heart Institute Journal.

2. Friedman, G. D., et al. (1974). White-blood-cell count as a predictor of myocardial infarction. New England Journal of Medicine.</td></tr>
</table>
