# Supplementary material for: Improvements in blood and fitness tracker biomarkers in a longitudinal real-world cohort of digital health platform users
Source: PLOS Digit Health. 2026 Mar 24;5(3):e0001271. doi: 10.1371/journal.pdig.0001271 (PMC13012459; doi:10.1371/journal.pdig.0001271)
Supplement: S1 Fig — (PDF) [file pdig.0001271.s012.pdf]

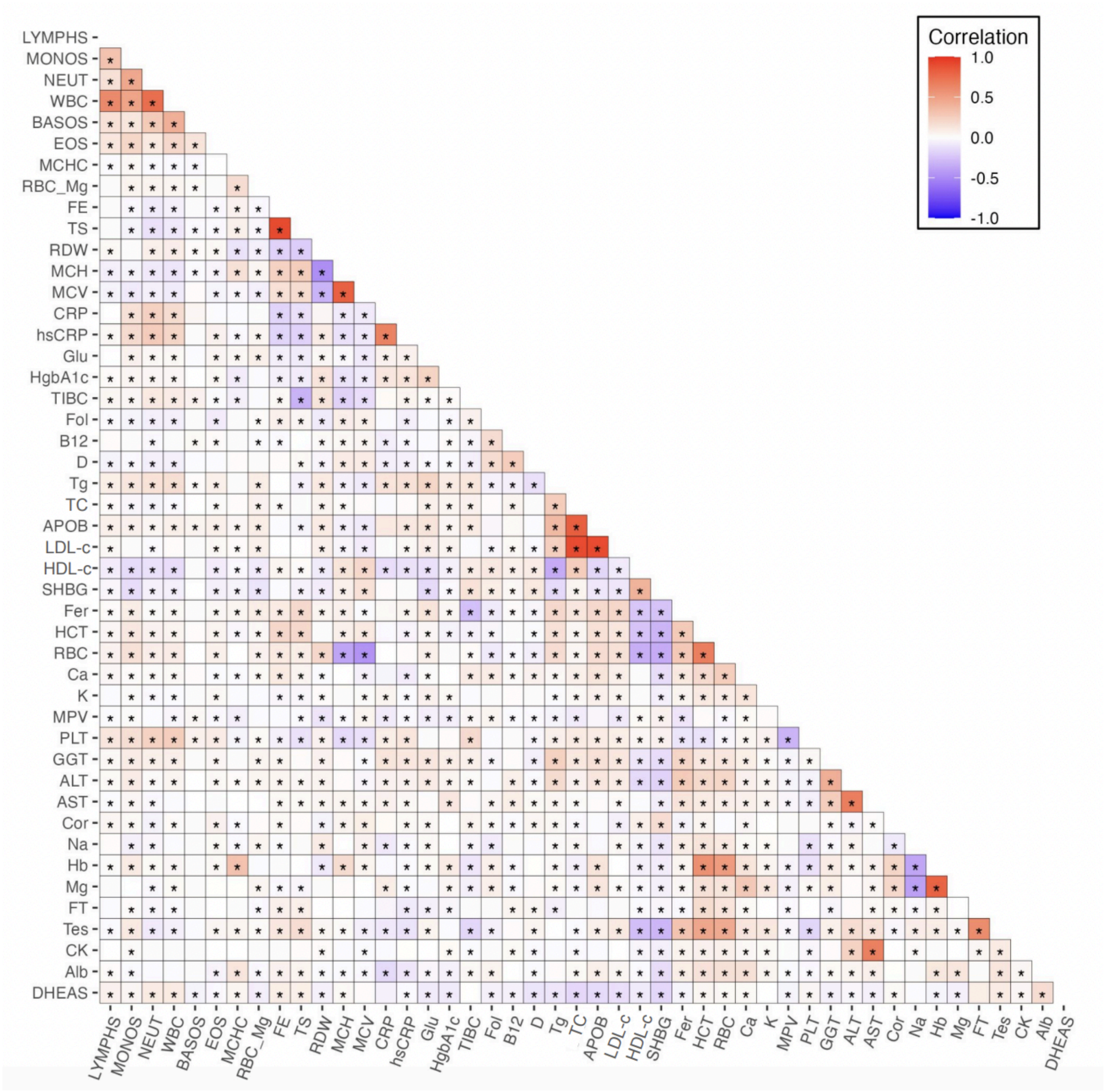

Figure S1. Biomarker cross-correlation among DHP users.
Heatmap of blood biomarker correlations in overall DHP user cohort shown. Colors correlate to the magnitude of Spearman correlation between baseline biomarker levels. Asterisks indicate multiple test-corrected p-values <0.05.
