# Supplementary material for: Improvements in blood and fitness tracker biomarkers in a longitudinal real-world cohort of digital health platform users
Source: PLOS Digit Health. 2026 Mar 24;5(3):e0001271. doi: 10.1371/journal.pdig.0001271 (PMC13012459; doi:10.1371/journal.pdig.0001271)
Supplement: S2 Fig — (PDF) [file pdig.0001271.s013.pdf]

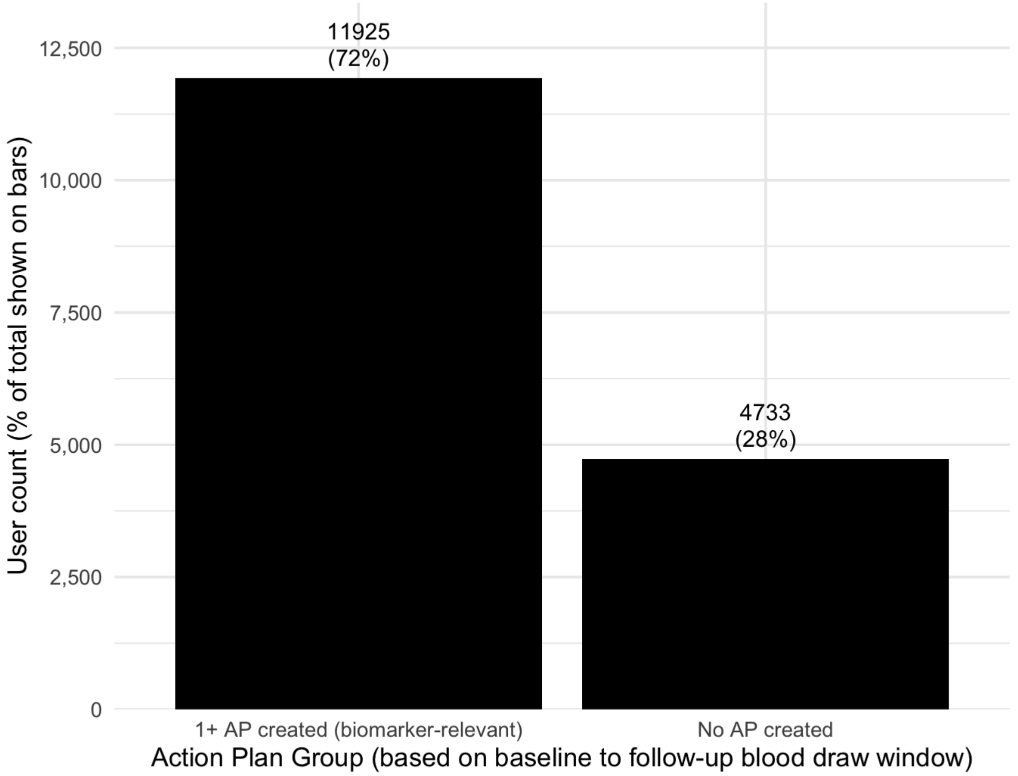

Figure S2. Action Plan adoption by DHP users. Number and percentage of users who created at least one biomarker-targeted Action Plan (1+ AP) between baseline and first follow-up blood draw compared with users who did not create an action plan (No AP) during this window. Data available for users with baseline blood tests from 2017-2023 (n = 16658).
