# Supplementary material for: Improvements in blood and fitness tracker biomarkers in a longitudinal real-world cohort of digital health platform users
Source: PLOS Digit Health. 2026 Mar 24;5(3):e0001271. doi: 10.1371/journal.pdig.0001271 (PMC13012459; doi:10.1371/journal.pdig.0001271)
Supplement: S3 Fig — (PDF) [file pdig.0001271.s014.pdf]

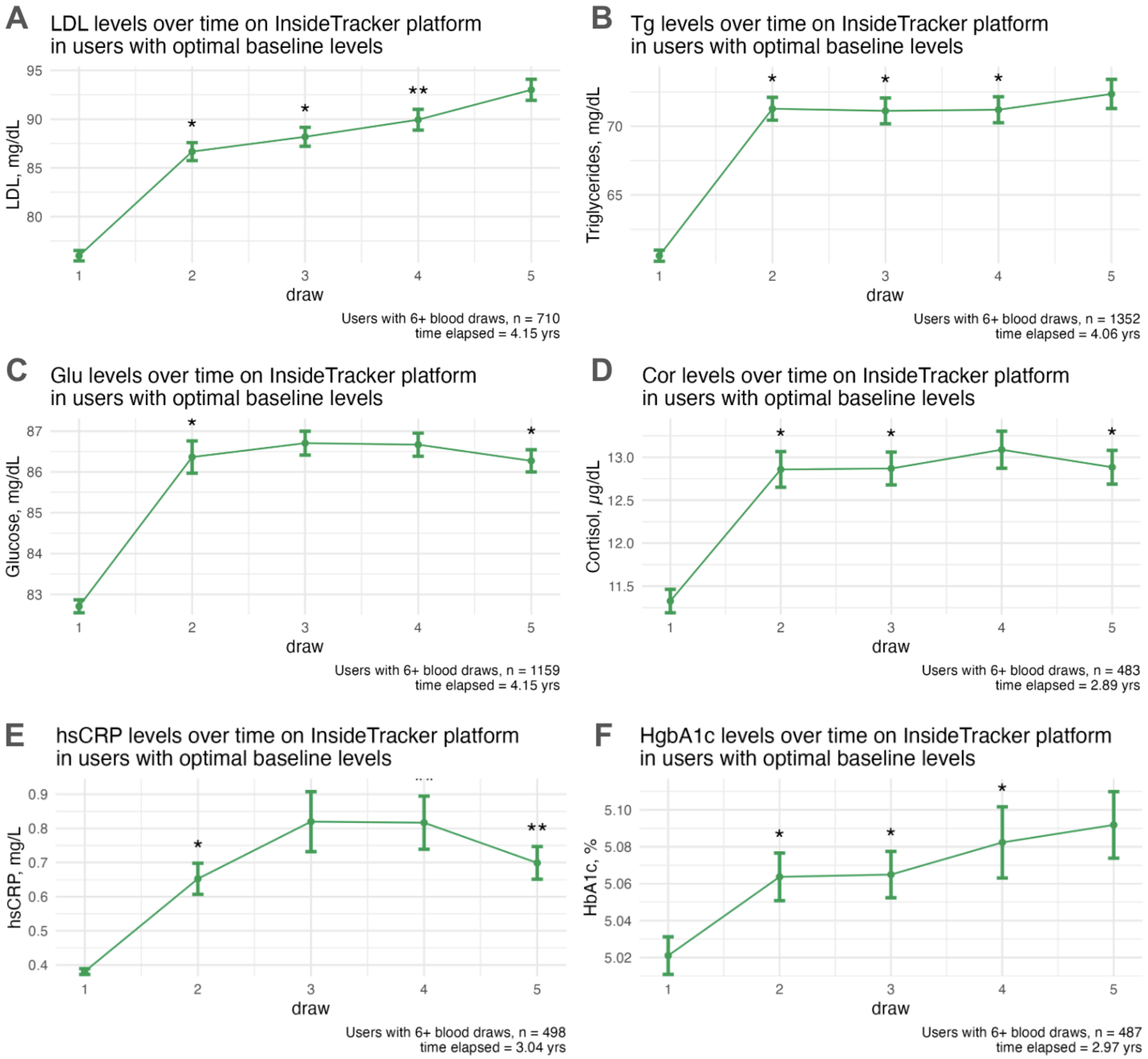

Figure S3. Long-term trajectories in biomarkers optimal at baseline (negative-exposure comparator). Biomarker levels for users are shown at baseline and at follow-up blood tests. **A**, LDL; **B**, Triglycerides; **C**, Fasted Glucose; **D,** Cortisol; **E** HgbA1c; **F,** hsCRP. A single asterisk indicates the mean value at that draw is significantly different from that of draw 1; two asterisks indicate the mean value at that draw is both significantly different from that of draw 1 and of the first follow-up draw (draw 2). Standard error bars and sample size are shown. User samples are matched throughout draws shown.
