# Supplementary material for: Improvements in blood and fitness tracker biomarkers in a longitudinal real-world cohort of digital health platform users
Source: PLOS Digit Health. 2026 Mar 24;5(3):e0001271. doi: 10.1371/journal.pdig.0001271 (PMC13012459; doi:10.1371/journal.pdig.0001271)
Supplement: S4 Fig — (PDF) [file pdig.0001271.s015.pdf]

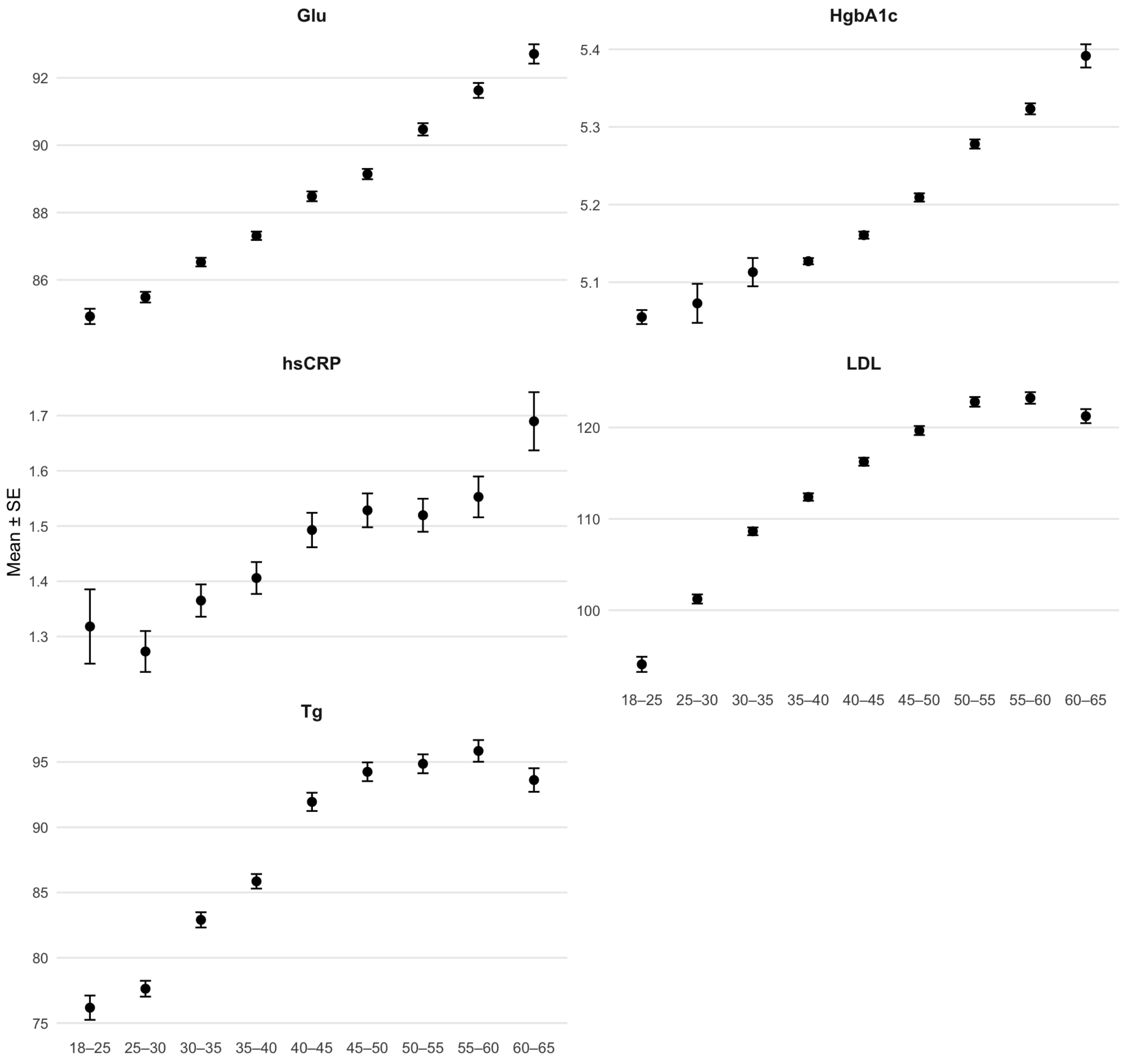

Figure S4. Age vs. biomarker level (mean ± SE) in 5-year bins. Baseline biomarker levels summarized by age for adults 18–65 years. The first bin is 18–25, followed by 5-year intervals. Points show the mean and bars show the standard error (SE) for each age bin. Per-bin n: 18–25 (1,489), 25–30 (3,971), 30–35 (6,348), 35–40 (7,126), 40–45 (6,451), 45–50 (5,280), 50–55 (4,814), 55–60 (3,587), 60–65 (2,574).
