# Supplementary material for: Improvements in blood and fitness tracker biomarkers in a longitudinal real-world cohort of digital health platform users
Source: PLOS Digit Health. 2026 Mar 24;5(3):e0001271. doi: 10.1371/journal.pdig.0001271 (PMC13012459; doi:10.1371/journal.pdig.0001271)
Supplement: S1 Methods — (PDF) [file pdig.0001271.s017.pdf]

Supplementary Methods: Statistical Analysis

All statistical analyses were performed in R using functions from the base `stats` package, with data manipulation conducted using standard tidyverse packages.

Outlier detection

```
analysis_df <- analysis_df %>%
  filter(
    value >= quantile(value, 0.01, na.rm = TRUE),
    value <= quantile(value, 0.99, na.rm = TRUE)
  )
```

Extreme values were excluded using percentile-based thresholds to reduce the influence of likely technical outliers.

Wilcoxon rank-sum tests

```
wilcox.test(
  biomarker_change ~ improvement_group,
  data = analysis_df,
  exact = FALSE
)
```

Nonparametric Wilcoxon rank-sum tests were used to compare distributions of biomarker change between groups, consistent with the non-normal distribution of several biomarkers.

Polygenic Risk Score (PGS) analyses

Polygenic risk scores were analyzed as categorical variables by stratifying individuals into low-, intermediate-, and high-risk groups based on population percentiles of the continuous PGS distribution. Associations between PGS-defined risk strata and longitudinal biomarker changes were assessed using analysis of variance (ANOVA), with post hoc comparisons performed where appropriate. Representative code illustrating these procedures is shown below.

```
# Assign PGS-based risk categories
analysis_df <- analysis_df %>%
  mutate(
    pgs_group = case_when(
      pgs_score <= quantile(pgs_score, 0.10, na.rm = TRUE) ~ "Low risk",
      pgs_score >= quantile(pgs_score, 0.90, na.rm = TRUE) ~ "High risk",
      TRUE ~ "Intermediate risk"
    ))

# Compare biomarker change across three PGS groups
anova_fit <- aov(biomarker_change ~ pgs_group, data = analysis_df)
summary(anova_fit)
```
