# Supplementary material for: Improvements in blood and fitness tracker biomarkers in a longitudinal real-world cohort of digital health platform users
Source: PLOS Digit Health. 2026 Mar 24;5(3):e0001271. doi: 10.1371/journal.pdig.0001271 (PMC13012459; doi:10.1371/journal.pdig.0001271)
Supplement: Attachment — Submitted filename: Response to Reviewers.docx [file pdig.0001271.s019.docx]

Nimisha Schneider

Senior Director, Science and AI, InsideTracker

[nschneider@insidetracker.com](mailto:nschneider@insidetracker.com)

August 19, 2025

Editor, PLOS Digital Health

Re: "Improvements in blood and fitness tracker biomarkers in a longitudinal real-world cohort of digital health platform users"

PDIG-D-25-00206

Dear Editor,

Thank you for the opportunity to revise and resubmit our manuscript. We appreciate the thoughtful and constructive feedback provided by the reviewers. Below, we provide a detailed, point-by-point description of the revisions made in response to each comment.

### **Reviewer 1 comments:**

1. **Request for explicit statement of study objective in abstract and main text**
 We added a clear statement of the study aim to both the abstract and main text to frame supporting background information in the introduction, and discussion of results:

“*In this retrospective, long-term longitudinal study of over 20,000 users of a digital health platform (DHP), we aimed to determine whether improvements in health-related biomarkers could be observed in users and, if so, how they were maintained over time. We further explored whether genetic predisposition and physiological patterns, such as sleep and activity, were associated with variability in these biomarker responses*.”

2. **Some tables lack clear legends or explanations for abbreviations used, and the quality of the figures is relatively low, making it difficult to interpret key details**

We added definitions for all abbreviations used in tables and increased figure text size for clarity. We can also provide higher-resolution figure files if needed for publication.

3. **Address the need to integrate these (Digital Health Platform (DHP)) tools with professional guidance, mitigating risks and ensuring ethical use.**

We added a description of the limitations of the tool in terms of its inability to substitute for medical care and its appropriate use by consumers in our Discussion section under Limitations, including descriptions of 1) ways that the tool encourages physician guidance in specific scenarios within the DHP, and 2) how clinical oversight from medical practitioners is encouraged in order to ensure the safety of users. E.g. “*We encourage integration of DHP outputs into conversations between users and their healthcare providers, explicitly instructing any user receiving a biomarker result that indicates emergent clinical risk to consult a physician. Given the clinical relevance of the biomarkers tracked in this product, usage of the DHP could be coupled together as part of routine care, under the guidance of a health professional in order to couple non-prescription to formal medical management*”.

### **Reviewer 2 comments:**

1. **Issues around lack of cohort gender and ethnic diversity and their implications**

The reviewer commented that a major study limitation is that the cohort lacks gender and ethnic diversity; they asked that we “include a thoughtful discussion of how the results could (or could not) apply to more diverse populations. This includes barriers such as cost, digital access, and cultural applicability. Discuss the practical accessibility of the platform for broader communities, especially in the Global South. Is the service affordable or adaptable for use in public health systems? If not, how could the insights still be useful? Consider including a statement on plans for future studies or platform adaptations that aim to improve inclusivity, if any.”

We fully agree that this is a major limitation and thank the reviewer for pointing out that it needs to be addressed thoughtfully. In response, we added a “Cohort Diversity” section to our Discussion under “Limitations”. This section includes a description of the cohort that specifically acknowledges study cohort issues in gender and ethnic diversity, and how we plan to expand our cohort of customers to mitigate lack of diversity in the future. We then address the potential issues with applying the results of studies done on skewed cohorts to a diverse population, giving referenced examples of health-related issues known to vary among groups defined either by race or ancestry. We further discuss the complexity of diverse study design based on the confluence or lack thereof of race and ethnicity, and how genetically defined ancestry may be used to address these complexities. We end this section by discussing ways in which the current study ***is*** applicable to a general and diverse population, as well as, to the extent which it is not applicable, some suggestions on how our results could be adapted for more relevance to diverse user groups including the Global South.

2. **Lack of a control group and limits to causal inference**

To address the point that our lack of a control group does not allow us to make causal conclusions in terms of our DHP impact on health, we expanded our Discussion to more clearly acknowledge that the absence of a control group limits causal interpretation. E.g. We frame the study as a hypothesis-generating, exploratory analysis and describe potential future directions for prospective, controlled validation studies.

3. **Justification of threshold choices**
To ensure that our choices of subcohort-defining thresholds are justified in a clear manner, we added the following information to our Methods section: For genetic risk, we explain the use of top and bottom decile cutoffs (T1/T3) and reference prior studies supporting this choice based on its phenotypic and clinical relevance in multiple conditions; for our definition of biomarker responders, we describe how our cutoffs correspond to clinical meaning, with a cholesterol-specific reference that is relevant to our “Characteristics of Users Who Improve Baseline Biomarker Levels Upon Retest***”*** results.

4. **Access to genetic data**
This reviewer asked us to address the lack of genetic data in the minimal dataset we provide, and how researchers wishing to replicate our analysis could potentially access this data. To the end of allowing other researchers to reiterate our genetic risk analysis, we added this to our data availability statement: “Access to the polygenic risk scores calculated from individual genetic data and used in this analysis may be requested from InsideTracker via the corresponding author for researchers who meet the criteria for access to confidential data.” Providing calculated genetic scores to researchers who can enter into a data sharing agreement with us will allow the analysis to be recapitulated without exposing individual genetic data at the raw genotype level.

5. **Transparency of DHP algorithm**
To address the request for additional transparency on the DHP algorithm logic, even in simplified form, we have added a description of the algorithm’s methodology to our methods section. We include an example of how a specific recommendation would be generated and a reference to a previous paper published by our group that provides additional platform algorithm detail.

### **Reviewer 3 comments:**

Reviewer 3 pointed out a lack of both a clear research question and of defined primary and secondary outcome measures; to address this concern, we specified our research question, aims, and primary and secondary outcomes as described above in response to Reviewer 1. This reviewer also commented that examining a dataset to make sense of it can be used only to formulate hypotheses, rather than constituting original research. Indeed, in our Discussion we acknowledge that observational, correlative studies are in an inherent sense simply generating causal hypotheses that must be tested in a prospective, controlled manner. However, we feel it is important to show, even in a correlative study, that observational evidence is consistent with health benefits for users of DHPs. Like many emerging tools in the rapidly evolving healthtech space, the examined DHP’s personalized recommendations are grounded in carefully curated, peer-reviewed evidence on nutrition, exercise, supplementation, and recovery. These recommendations are assumed to benefit individual users based on their biological profiles, but assumptions alone are insufficient. We believe it is essential for us, as well as other commercial wellness enterprises, to undertake studies that assess real-world patterns and outcomes among our actual users. While of course rigorous, controlled trials will be required to fully evaluate efficacy and causality of DHPs, we believe such cohort-based studies are a meaningful and scientifically important first step.

We greatly appreciate the reviewers’ feedback and believe these revisions strengthen the clarity, transparency, and rigor of our manuscript. Thank you for considering our revised submission. We look forward to your response.

Sincerely,

Nimisha Schneider

(on behalf of all co-authors)
