# Supplementary material for: Improvements in blood and fitness tracker biomarkers in a longitudinal real-world cohort of digital health platform users
Source: PLOS Digit Health. 2026 Mar 24;5(3):e0001271. doi: 10.1371/journal.pdig.0001271 (PMC13012459; doi:10.1371/journal.pdig.0001271)
Supplement: Attachment — Submitted filename: Response letter, revision 2 PLOSDH 11-4-2025.docx [file pdig.0001271.s020.docx]

Nimisha Schneider

Senior Director, Science and AI, InsideTracker

[nschneider@insidetracker.com](mailto:nschneider@insidetracker.com)

November 9, 2025

Editor, PLOS Digital Health

Re: "Improvements in blood and fitness tracker biomarkers in a longitudinal real-world cohort of digital health platform users"

PDIG-D-25-00206R1

Dear Editor,

Thank you for the additional opportunity to revise and resubmit our manuscript. We appreciate the questions raised in response to our first revised manuscript and have endeavored to address them thoroughly. Below, we provide a detailed, point-by-point description of the revisions and additions made to our resubmitted manuscript in response to each comment.

1) *Question: The levels of compliance among the users may be a key issue. How about the compliance of the users? How did you assess the level of user compliance? Did the platform have any specific way to improve compliance/cope with low compliance?*

We agree that user compliance is central to interpreting real-world effectiveness of the lifestyle recommendations in the DHP. In the present retrospective analysis we did not directly measure per-user compliance with specific lifestyle recommendations between tests. We do not currently collect that data from users in a systematic manner. The only exposure of the cohort in this correlative analysis we can systematically assert is the informational intervention (personalized results and education delivered with baseline blood biomarker results). However, to partially address the compliance question, we added two complementary analyses:

1. **Possible, partial proxy for compliance: behavioral engagement**

Our platform includes an “Action Plan” (AP) feature: users examine the list of recommendations they receive matching their baseline biomarker profile, and choose a shorter list of specific recommendations that best fit their personal goals, lifestyle and habits to follow for 90 days. During this time period, they receive in-app reminders and behavioral nudges. While not compliance data, engaging with the behavioral health aspect of the DHP could reasonably be projected to correlate with increased recommendation compliance, at least to some extent.

In a stratified analyses of biomarkers of interest (cohorts with higher vs lower than optimal marker levels at baseline analyzed separately, allowing improvement outcomes to be assessed in a single direction of better health), users who engaged with APs targeting a given biomarker showed greater improvement in that biomarker at the first follow-up blood draw than a comparison cohort of users with no APs at all in the baseline to follow-up window. We evaluated categorical zone transitions (improvement ≥1 zone) using 2×2 contingency tables and Pearson’s χ² or Fisher’s exact tests, as appropriate, with multiple-testing control (tables shown below and added to supplementary data (Tables S6a and b).

**Zone-transition outcomes by cohort**

***Action Plan targeting biomarker vs no Action Plan; biomarkers above optimal at baseline***

AP = Biomarker-relevant Action Plan created by user in the baseline to follow-up blood draw window;

noAP = no Action Plan created by user in the baseline to follow-up blood draw window.

‘met’ = achieved the specified outcome (improved ≥1 zone).

Negative risk differential indicates greater outcome success in AP cohort.

**Zone-transition outcomes by cohort**

***Action Plan targeting biomarker vs no Action Plan; biomarkers below optimal at baseline***

AP = Biomarker-relevant Action Plan created by user in the baseline to follow-up blood draw window;

noAP = no Action Plan created by user in the baseline to follow-up blood draw window.

‘met’ = achieved the specified outcome (improved ≥1 zone).

Negative risk differential indicates greater outcome success in AP cohort.

However, we understand that while these analyses are informative and allow us to hypothesize in a more informed manner about the role of compliance, particularly in improving key biomarkers including LDL, HgbA1c, and Vitamin D (significantly higher proportion of users improved these markers by 1+ zones when a biomarker-relevant AP was created), they cannot be extrapolated to causal inference as currently designed. First, cohort assignment (creation vs. non-creation of a biomarker-specific AP after the baseline test) reflects user self-selection rather than randomized assignment and may reflect unmeasured determinants (e.g., care access, other material resources, time affluence, etc.) that constitute potential confounding variables. The cohorts are also associated with baseline biomarker differences (SMD plots showing these imbalances are included below), which may influence improvement outcomes. Additionally, we know from user interviews that many individuals who do not engage with the AP feature of the product are actually participating in high-intensity health interventions external to our product (e.g., utilizing a concierge medical service, personal trainer, and/or nutrition coach or dietician.) These users harness our product by downloading all the recommendations provided to them and engaging with them on their own. In their specific cases, lack of engagement with our digital action plan feature is not correlated with lack of engagement with the health recommendations or lack of compliance with them. Since we do not collect this kind of data about users, we only have anecdotal evidence of this behavioral phenomenon among a subset of our users and cannot easily quantify it within an analysis.

Lastly, note that AP data for users is not available for dates before 2017; our engagement correspondence with improvement analysis therefore covers most but not all of the user cohort included in our larger study.

Despite these considerations, we understand that any information about user compliance, even indirect and observational, is of great interest to potential readers of our work and have included the supplementary engagement analysis results as well as a brief discussion of the above points in the revised version of the publication.

2. **Indications that compliance is relatively high in the overall DHP user cohort**

We have a couple of ways we can address the issue of estimated overall cohort compliance:

Continuing to treat AP engagement as a proxy for increased recommendation compliance: we observe that AP uptake was high across the total user cohort; ~72% of users with 2+ blood tests created at least one AP in the baseline to follow-up blood draw window, suggesting broad engagement with the behavioral component of the platform and extrapolating to relatively high compliance of the overall cohort. A plot is shown below and has been added to our Supplementary Data submission (Figure S2).

Compliance levels by self-report: In a small follow-up user survey (limited *n*; 53 users), 87% of respondents reported implementing one or more recommended interventions between tests. We understand that not only is this sample size very small compared to our overall cohort, but survey instruments are highly susceptible to selection and nonresponse bias; it is plausible that users who are willing to complete a follow-up survey are systematically more engaged and adherence-prone than non-respondents, for example. The self-reported compliance rate may overestimate true compliance in the broader user population. However, it is an interesting data point which allows us to hypothesize relatively good levels of compliance in our cohort and we have now briefly mentioned it in our Discussion of adherence in the cohort.

Evidence from other published studies does support the notion that exposure to information and education on lifestyle and dietary changes can yield positive health outcomes, presumably through a mechanism of enhanced adherence to those recommendations. For example, a meta-analysis of studies examining the effectiveness of nutritional education and recommendations from a dietician in adults with dyslipidemia showed that the educational intervention alone (compliance not measured) resulted in significant improvements in total cholesterol, LDL cholesterol, triglycerides, and HgbA1c^1^. The interventions examined in this study are not identical to the lifestyle recommendations provided to the users in our study, as they involved in-person meetings with a dietician, but do support the idea that healthy lifestyle education alone can result in blood biomarker improvements. Another study looked at the effect of providing reading material containing recommendations on diet and lifestyle changes to help with glycemic control in Type 2 Diabetics, and showed that this informational intervention was linked to significantly larger improvements in fasted glucose, triglycerides, and both LDL and HDL cholesterol^2^. We have added these references and a brief discussion of their relevance to our study to our publication (Discussion section).

2) *Question: Causal inference is a major concern of the reviewers. For causal inference, Mendelian randomization may be used: you have polygenic risk scores (PGS) as instrumental variables, BMI/LDL-c/… as exposure variables, the changes in the values as outcome variables, and the levels of compliance as disturbance variables (if the levels of compliance were not the same for all users). This is also for what you mentioned in lines 78-79: to assess whether genetic risk influenced the degree of improvement.*

Regarding the suggestion to use Mendelian randomization (MR) with biomarker polygenic scores (PGS) as instruments: given our dataset, we believe the specific MR analysis suggested is not feasible to address the specific causal question at hand, i.e., the effect of the DHP’s health recommendations/lifestyle behavior component on change in biomarkers, due to both practical and conceptual considerations:

The proposed design would primarily test whether baseline biomarker levels (exposure) causally impacts subsequent biomarker change (outcome) and whether compliance alters that pathway. However, that exposure impact on outcome described in this way is likely to be weak or absent for many biomarkers. As we report in our results section *Genetic Predisposition and Biomarker Changes*, for many biomarkers, a higher baseline level does not correlate with greater subsequent improvement/decline in a way consistent with the MR setup. While our results highlight the finding that a relationship exists for a few markers, even in these cases it is likely not the only mechanism influencing change (other behaviors and physiology contribute), so the exclusion pathway required for MR is probably insufficient.

Even if we take the proposed model design at face value, the available PGS explain only some of the variance in baseline levels (e.g., ~5–6% for LDL; ~12% for MPV; see Table 2, *Correlation between polygenic traits and baseline blood biomarker levels*, in our Results section), which likely do not constitute strong enough instruments for stable MR estimates, especially when the outcome is a change score.

Finally (and most directly relevant), because compliance was not directly measured in the present study data, we cannot use it as a moderator in the model as proposed. Using platform engagement as a partial proxy for compliance (as described above) would add measurement noise; combined with the lack of instrument strength and weakness of the instrument/exposure-outcome link, we feel that an MR analysis with the study dataset as is would not provide an interpretable result.

However, we understand the problem of establishing causality in our data as one of major concern. We have framed our results as associations that, without a prospective study including proper randomization and controls, serve merely as hypotheses-generating when it comes to causality. The major weakness precluding causal inference in our study is our lack of a control group; therefore, we have added two supplementary analyses which can address this issue of controls:

1. **Negative-exposure comparison**

While our dataset lacks a true intervention-free control, we can approximate a negative-exposure comparison using a cohort of users with extensive longitudinal data and had optimal baseline levels for a given biomarker (who therefore received no recommendations for that biomarker). For several key markers, we observe a slow and modest upward drift over time, where levels that start optimal become slightly less so (although most users remain within optimal or normal zones). In contrast, users who began above optimal (and thus received recommendations) show sustained improvements (Figure 2 in Results section, *Long-term user trajectory of improvement in non-optimal at baseline biomarkers*), which is consistent with an intervention effect. Notably, the drift among optimal-at-baseline users attenuates after the second draw, likely because some individuals cross out of the optimal zone and subsequently receive targeted recommendations, which may curb further deterioration. We recognize that recommendations are not independent across biomarkers: interventions aimed at one domain (e.g., improving elevated fasting glucose via diet/activity) can impact others (e.g., LDL), complicating a clean attribution but further supporting the plausibility of behavior-mediated improvements. By design, this negative-exposure cohort differs at baseline from the interventional cohort correlated with improvement in our main study (optimal vs above-optimal biomarker levels). This constitutes a potential confounding variable, and the cohort is not meant to be treated as a counterfactual for the intervention group. Rather, it provides supplementary context on the behavior of biomarkers under minimal platform exposure for that biomarker (no targeted guidance), supporting the plausibility that targeted recommendations contribute to the sustained improvements observed in users who start at suboptimal biomarker levels. The negative-exposure trajectory plots are below and have been added to our Supplementary Data submission (Figure S3), and their value briefly discussed in the causality section of *Limitations* within our Discussion. We have additionally cited a relevant study comprising a historical control group where HgbA1c was measured over several years in a general US population (no intervention in the study design), and modest, slow increases were observed over time.

**Long-term trajectories in biomarkers optimal at baseline (negative-exposure comparator).** Biomarker levels for users are shown at baseline and at follow-up blood tests. **A**, LDL; **B**, Triglycerides; **C**, Fasted Glucose; **D,** Cortisol; **E** HgbA1c; **F,** hsCRP. A single asterisk indicates the mean value at that draw is significantly different from that of draw 1; two asterisks indicate the mean value at that draw is both significantly different from that of draw 1 and of the first follow-up draw (draw 2). Standard error bars and sample size are shown. User samples are matched throughout draws shown.

2. **Cross-sectional biomarker trajectories by age**

As additional context for negative-exposure biomarker trends, we examined age trajectories for specific key biomarkers in cross-section from users’ baseline levels. Shown below and added to our Supplementary data (Figure S4), these markers exhibit a gradual drift in the direction of worse health with age. Although cross-sectional, these age gradients are consistent with the longitudinal negative-exposure finding above, that without intervention, key biomarkers do not trend in the direction of improvement. Both the age trajectories and the negative-exposure longitudinal trajectories lend some support to the hypothesis that targeted recommendations contribute to the sustained improvements observed in DHP users who start at suboptimal biomarker levels.

3) *Question: Please highlight unique findings in the paper. What findings in this study have not been mentioned in other studies?*

The scale, integration of multiple modalities of health data (blood biomarkers, activity and sleep data, and DNA), and long-term longitudinal analyses distinguish this work from prior reports and move the literature beyond marketing claims toward peer-reviewed evidence of DHP-associated changes in health. To our knowledge, this is the first study to show that DHP-correlated improvements in clinically accepted blood biomarkers (e.g., LDL-C, HbA1c, micronutrients) are sustained over years and multiple retests. Additionally, though other studies have observed genetic risk (PGS) correlations with baseline phenotypes, we here show their link to subsequent change for specific biomarkers (e.g., individuals with higher lipid-risk PGS exhibit less cholesterol improvement). Though this genetics-to-response effect size is small and the breadth of the phenomenon across multiple biomarkers is limited, additional sample size power may allow us to expand this preliminary finding in the future and uncover more about the impact of genetic risk on lifestyle-induced health improvements. Lastly, though the cardiovascular health impacts of activity and sleep quality are well-established in the literature, we here link simple, interpretable behavioral signals from wearables (nightly REM sleep percentage and an average increase of approximately 1000 daily steps between tests) to the ability of DHP users to shift cholesterol profiles in a healthier direction, offering practical behavioral targets beyond cross-sectional associations. We have revised the *Impact* subsection of our Discussion to explicitly highlight these unique contributions as context for the reader.

Once again, thank you for your feedback and the opportunity to edit and expand our manuscript. We are happy to answer any additional questions or provide additional data. We look forward to your response.

Sincerely,

Nimisha Schneider

(on behalf of all co-authors)

References

1. Sikand G, Handu D, Rozga M, de Waal D, Wong ND. Medical Nutrition Therapy provided by dietitians is effective and saves healthcare costs in the management of adults with dyslipidemia. Curr Atheroscler Rep. 2023;25(6):331-342.
2. Gortzi O, Dimopoulou M, Androutsos O, Vraka A, Gousia H, Bargiota A. Effectiveness of a nutrition education program for patients with type 2 diabetes mellitus. Appl Sci. 2024;14:2114.
