# Supplementary material for: Improvements in blood and fitness tracker biomarkers in a longitudinal real-world cohort of digital health platform users
Source: PLOS Digit Health. 2026 Mar 24;5(3):e0001271. doi: 10.1371/journal.pdig.0001271 (PMC13012459; doi:10.1371/journal.pdig.0001271)
Supplement: Attachment — Submitted filename: Response letter, revision 3 PLOSDH 2-4-2026.docx [file pdig.0001271.s021.docx]

Nimisha Schneider

Senior Director, Science and AI, InsideTracker

[nschneider@insidetracker.com](mailto:nschneider@insidetracker.com)

February 4, 2026

Editor, PLOS Digital Health

Re: "Improvements in blood and fitness tracker biomarkers in a longitudinal real-world cohort of digital health platform users"

PDIG-D-25-00206R2

Dear Editor,

Thank you for the additional opportunity to revise and resubmit our manuscript. We appreciate the questions raised in response to our second revised manuscript and have endeavored to address them thoroughly. Below, we provide a detailed, point-by-point description of the revisions and additions made to our resubmitted manuscript in response to each comment.

**Major points of revision:**

**“The interpretation of sustained improvements across >5 draws still risks regression-to-the-mean inflation; the authors acknowledge this, but could further quantify the likely magnitude of this effect.”**

To address the issue of regression to the mean (RTM) explicitly, we added an analysis to estimate RTM-attributable change within the longitudinal analysis. RTM is expected to primarily impact the baseline to draw 2 biomarker changes; RTM effects were therefore estimated for first follow-up change (baseline draw to draw 2). We calculated the per-biomarker baseline population mean $\mu$ (rather than the mean of the suboptimal (in the direction of worse health) cohort used for longitudinal improvement analysis) and the population-level correlation between draws 1 and 2 $\rho₁,₂$ , and used this to estimate the possible RTM-attributable change in the analysis cohort as $(1-\rho₁,₂)(\mu-x₁)$. We then calculated the percent of the mean changes observed in our longitudinal analysis that could be attributed to RTM. These data are presented in Table S7. We see that all markers have a below 100% RTM contribution estimated, but the contribution varies greatly across biomarkers. Those with the greatest RTM impact are markers with higher biological variation and technical variation, as expected. For example, both cortisol and glucose are subject to variation based on blood draw time and fasted status.

We observed two longitudinal biomarker trajectory patterns in our data (Figure 2): For LDL, HDL, triglycerides, vitamin D, vitamin B12, and testosterone, biomarker values continued to improve in long-term DHP users beyond the second draw. For other biomarkers examined, the majority of improvement occurred between baseline and the second draw and was then maintained for multiple years. This pattern is consistent with rapid early changes (e.g., initiation of supplementation or lifestyle modifications) followed by stable maintenance, and is not expected under a pure regression-to-the-mean artifact, which would not produce durable multi-year stabilization at improved levels in the presence of ongoing measurement variability.

We have added text to our discussion section pointing to the RTM analysis results in the supplement and commenting on the variation in RTM contribution among biomarkers. We have also added an outline of the technical approach in the methods section.

**“The “optimal zone” definition, although better described, still mixes clinical reference ranges with proprietary definitions; greater clarity would strengthen reproducibility. Provide a supplementary table listing the rationale and sources for each biomarker’s “optimal” band, distinguishing clinical vs internal definitions.”**

Since we use “suboptimal” as a cohort-defining filter at multiple points in our analysis, we appreciate the need to clarify the derivation of our optimal zone and peer-reviewed sources that support it on a per-biomarker basis. We have added **Supplementary Table S8: Optimal Zones Documentation** containing this information. This is uploaded as a separate supplementary document due to its length. We added reference to this table in the methods section where zones are described.

**Minor points of revision:**

**Clarify whether the “minimal dataset” includes all variables used for statistical testing:** Yes, the minimal dataset includes all needed variables including wearables data. We added a short description to the supplementary files list.

**The small n for the wearable-sleep subgroup (n=126) and relaxed p-value threshold (0.1) should be highlighted more prominently as exploratory:** We have clarified the limitations around this particular analysis with additional text in the discussion: “These wearable-associated analyses characterizing TC improvers should be considered exploratory. The subgroup of users with available fitness tracker and sleep data was relatively small (n = 126), and a relaxed significance threshold (α = 0.1) was applied to balance limited statistical power against the substantial day-to-day variability of wearable-derived measures. As such, these findings are best interpreted as preliminary signals that motivate further investigation, rather than as definitive evidence, and will be reassessed as larger wearable-enabled cohorts become available.

**Ensure all language aligns with associational findings (e.g., “correlates with” rather than “impacts” or “leads to”):** We have made several changes to the language used in the manuscript, mainly in the discussion, to ensure this alignment.

**Code transparency (if feasible) - Include code snippets or analytic scripts used for the Wilcoxon analyses, PGS stratification, and outlier detection, even if only pseudocode.** We have added a Supplementary Methods section to the main supplementary data file providing representative code snippets illustrating the Wilcoxon analyses, polygenic score stratification and ANOVA, and outlier handling procedures, using standard R statistical functions.

**Add one or two sentence highlighting limitations of ancestry mismatch (CEU proxies) and potential bias for non-European users:** We have added the following text to our limitations section: “Because proxy variants for missing SNPs were derived using linkage disequilibrium patterns from a European-ancestry reference population (CEU), polygenic risk score estimates may be less accurate for users of non-European ancestry. As a result, associations involving PGS should be interpreted with caution in these groups and may underestimate true effects.”

**Line edits:**

**Lines 36-37:**

**users who increased their daily steps by ~1,000 and those with higher REM sleep percentages were more likely to shift cholesterol in a healthier direction.**

**—However, Figure 5A shows that at some time points, the difference between the two groups is far greater than 1,000 steps？**

Indeed, there is an apparent discrepancy between the magnitude of step count differences shown at individual time points in Figure 5A and the summary value reported in the text. We have revised the manuscript to clarify that the reported ~1,000-step difference refers to the average increase in daily step count from baseline to post-baseline follow-up, rather than to pointwise differences at individual monthly time points. We also added explicit acknowledgement of the substantial month-to-month variability in step counts observed among improvers, as shown in Figure 5A. These lines now read “Data from wearables provided information on who improved: users who increased their daily steps by an average of ~1,000 steps from baseline to follow-up activity levels, and those with higher REM sleep percentages, were more likely to shift cholesterol in a healthier direction, despite substantial month-to-month variability in step counts.”

**Lines 164-169：**

**A high proportion of users were able to improve HgbA1c, fasting glucose, triglycerides, and hsCRP levels (79.3%, 74.2%, 76.2%, and 70.7% respectively; Figure 1E, C, B, F), but relatively few could completely optimize them (21.4%, 31.1%, 29.6%, and 25.4%, respectively, of suboptimal users improved).**

**LDL-c also showed this pattern: a relatively higher proportion (20.4%) of users able to improve vs (5.3%) optimize their initially high levels by their follow-up blood test.**

**— What are the differences between these two groups (improve vs. completely optimize)—behavioral differences, differences in Polygenic Risk Scores, or others?**

Characterizing users who improve biomarker levels versus those who fully optimize them is an interesting area for future investigation. However, at present, we are limited by data availability and cohort size for such analyses. Only a small subset of the longitudinal cohort has appropriately linked wearable data, and further stratifying this group into “improvers” versus “optimizers” would leave us lacking statistical power for proper analysis, particularly given the general noisiness of wearable-derived measures. Similarly, polygenic risk score data are available for fewer than half of users in the longitudinal cohort, which precludes robust stratified characterization analyses between these subgroups at this time. We do hope to examine characterization of full optimizers in the future additional data accrue, particularly as we collect larger quantities of wearables data.

**Lines 190-194:**

**The ferritin PGS most significantly associated with longitudinal changes with the corresponding serum biomarker (Figure 4C): high baseline ferritin levels decreased on average upon retest in T1, T2, and T3 users; however, the decrease was significantly larger for users in T1 than those in T2 or T3.**

**—This analysis examined the association between TC levels and physical activity, but no PGS data were available. Does the effect of physical activity on TC levels depend on an individual's PGS?**

See above; it is sample size limiting to filter a cohort of users who have both genetics data, longitudinal blood cholesterol levels, and appropriately time-linked wearables data (the latter being the most limiting factor). Unfortunately we are not yet powered to do this analysis, even with a very large longitudinal blood data cohort. It is definitely of interest to us, since the analysis could pave the way to hypothesizing about whether physical activity can limit negative polygenic risk, e.g. for hyperlipidemia, in any way.

Once again, thank you for your feedback and the opportunity to edit and expand our manuscript. We are happy to answer any additional questions or provide additional data. We look forward to your response.

Sincerely,

Nimisha Schneider

(on behalf of all co-authors)
